# *Plasmodium falciparum* Chloroquine Resistance Transporter (PfCRT) Is a Redox-Dependent Drug Transporter

**DOI:** 10.1021/acs.biochem.5c00802

**Published:** 2026-03-11

**Authors:** Darius Chernitsky-Hamd, Rajat Roy, Jonas Schemm, Aubrey Schall, Mera Petros, Andreas Willems, Paul D. Roepe

**Affiliations:** Dept. of Chemistry and Dept. of Biochemistry and Cellular and Molecular Biology, 8368 Georgetown University, 37th and O Streets NW, Washington, D.C. 20057, United States

## Abstract

The recently elucidated atomic resolution cryo–EM structure of the 7G8 isoform of *Plasmodium falciparum* chloroquine resistance transporter (PfCRT) suggests two pairs of proximal cysteine residues within the loop 7 (L7) domain. We wondered whether these might provide a redox active switch that might then regulate PfCRT function. Using site-specific mutagenesis, maleimide labeling, redox buffering, and chloroquine transport measurements, as well as molecular dynamics (MD) calculations, we probe the relative importance of all Dd2 PfCRT isoform C as well as their HS SH to S–S interconversion vs CQ transport function. Results show that CQ transport by PfCRT is regulated by the redox potential. We propose that disulfide bonds form at both the C289/C312 and C301/C309 pairs of Dd2 PfCRT and that these dynamic S–S bonds are required for full PfCRT CQ transport activity. Mutagenesis of all Dd2 PfCRT C to S or A reveals that no other C is functionally obligate but identifies C101, C139, C171, and C328 as involved in modulating CQ transport. Since two of the L7 C (C309 – C312) are within a CXXC motif (with X = D) that in theory can signify a metal binding site, we also model divalent metal ion binding using Metal3D and AlphaFold 3 and find that divalent metal may coordinate to C elsewhere in the protein but likely not to this CXXC motif. MD calculations done with 10 ns or 1 μs trajectories suggest large conformational changes in L7 near the initial drug binding site upon SH HS to S–S interconversion. Together, the data yield a model for how L7 disposed to the redox active digestive vacuole (DV) of the intraerythrocytic malarial parasite regulates PfCRT access to DV-disposed CQ^2+^.

## Graphical abstract

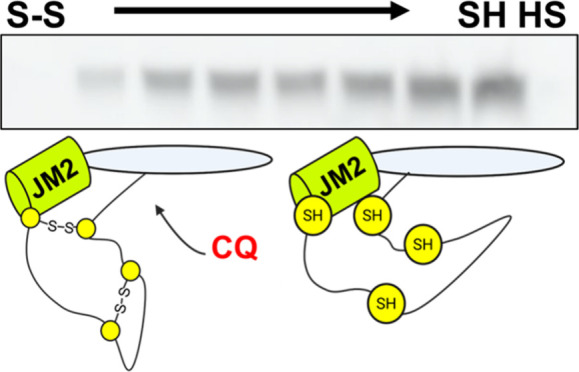

## Introduction

The *Plasmodium falciparum* chloroquine resistance transporter (PfCRT)^1^ is found within the membrane of the intraerythrocytic malarial parasite digestive vacuole (DV) and includes a CDDC motif that could signify a metal binding site.^2,3^ The DV is a lysosome-like organelle that digests copious host red blood cell (RBC) hemoglobin (Hb) and crystallizes concomitantly released toxic ferriprotoporphyrin IX (FPIX) heme^4^ to inert hemozoin (Hz) during rapid intraerythrocytic growth of the parasite within the infected RBC (iRBC). When mutated to specific isoforms found within chloroquine-resistant (CQR) parasites, PfCRT mediates increased DV lumen to parasite cytosolic transport of charged chloroquine (CQ) in a membrane potential and ΔpH-dependent fashion^5–9^ to confer cytostatic CQ resistance (CQR). Toxic FPIX to inert Hz crystallization is inhibited by CQ and other drugs and is highly redox (reductive/oxidative environment)-dependent. In the live DV-localized catabolism of Hb and concomitant release of FPIX heme, superoxide, peroxyl and hydroxyl radicals must be scavenged to avoid cell damage.^10^ Thus, DV redox is profoundly important for the parasite life cycle, as well as the molecular pharmacology of many antimalarial drugs. We have recently measured that plasma levels of quinoline or artemisinin-based antimalarial drugs cause a significant oxidative “burst” within the DV.^10^ Resistance to artemisinin-based prodrugs, a principal component of ACTs (artemisinin combination therapies), has so far only been observed in CQR malaria expressing a mutant PfCRT isoform.^11^ Potency of these prodrugs relies on conversion to their active drug form by reductive cleavage of an endoperoxide bridge, which also depends on the redox potential of the DV.^12–14^

Therefore, the DV struggles with redox regulation that influences the potency of DV-acting antimalarial drugs. Indeed, several earlier studies^15–19^ point to the potential involvement of DV redox chemistry in antimalarial drug resistance phenomena. How CQR-conferring isoforms of PfCRT, which are necessary for the most common form of antimalarial drug resistance, might respond to DV redox has not yet been explored.

The three-dimensional atomic resolution structure of PfCRT reveals two pairs of proximal cysteine residues within the DV-disposed loop seven (L7) connecting helices seven and eight.^1^ The redox potentials of lysosome or lysosome-like organelles (e.g., the DV) have not yet been well studied, but since the organelles are acidic, lysosomal lumen is expected to be reducing, as we have measured for the live intraerythrocytic parasite DV.^10^ How DV redox might affect PfCRT disulfides is not yet known. Several other membrane transporters have been shown to harbor redox sensitive cysteines that convert free thiols to a disulfide bond as part of a “redox sensing switch” that regulates transporter activity.^20–27^ For example, a disulfide bridge between residues C180 and C329 regulates transport activity of the human amino acid transporter hPAT1 ^20^ and a C249–C321 bridge regulates the SNAT4 amino acid transporter.^27^ We thus wondered if PfCRT L7 proximal C289/C312 and C301/C309 pairs, whose thiol groups are defined by MD and cryo-EM to be close enough to possibly form S–S bonds,^28,29^ might exhibit disulfide-free sulfhydryl interconversion at physiologically meaningful redox potentials, and if so, whether this might regulate PfCRT CQ^2+^ transport activity.

## Materials and Methods

### Chemicals and Reagents

DTT (dithiothreitol) was supplied by GoldBio (CAT: DTT10) and cyclo-DTT (trans-4,5-dihydroxy-1,2-dithiane) was supplied from AdipoGen (CAT: CDXD0277G001). ßME (2-mercaptoethanol) was supplied by Sigma Aldrich (CAT: M6250), and the ßME-dimer (2-hydroxyethyl disulfide) was supplied by Sigma Aldrich (CAT: 380474). Liquid ßME reagent bottles were stored at 4 °C and were purged with nitrogen after each opening. 100 mM stock solutions were prepared periodically and stored at −80 °C. Stock solutions were thawed on ice in the dark until preparation of redox buffers. TCEP (tris­(2-carboxyethyl)­phosphine) was supplied by GoldBio (CAT:TCEP1) as the HCl salt. Powders were stored in parafilm sealed desiccators at −20 °C. Samples were allowed to warm to room temperature prior to opening to prevent condensation.

### Yeast Expression Plasmids

The plasmids pYES2 PfHB3-V5–6xHIS (VH) and PfDd2-VH were constructed previously and used to synthesize improved constructs encoding expanded epitope labeling at the C terminus of expressed PfCRT.^5,6,30^ The tag-optimized pYES2 plasmids were then used as template for oligonucleotide site-directed mutagenesis via the Q5 method.^31^

### Mutagenesis

The Q5 Polymerase and Hot Start Master Mix (New England Biolabs [NEB]) were first used in a PCR reaction to loop-in 6 additional HIS codons prior to our previous 3′-encoded tag.^5,6^ The same procedure was repeated to loop in a Tobacco Etch Virus (TEV) [GAGAACCTGTACTTCCAGGGC] protease site between the V5 epitope and 12× HIS tag, to provide an in-frame tag (Table S1) in the final *pfcrt* construct that we call “V5-TEV-12xHIS” (Vt12H).

This was done to improve purity of dodeca-his-*pfcrt* when eluted from Ni^2+^ or Co^2+^ chelating resin beads in the presence of detergent (see Results). Mutant *pfcrt* isoforms detailed in Table 1 were then created using the pYES2 Dd2-Vt12H and pYES2 HB3-Vt12H plasmids and the Q5 Site-Directed Mutagenesis Kit (NEB) according to manufacturer instructions. Back-to-back forward and reverse primers were designed using the ″NEBaseChanger″ tool (Table 1).^32^ The PCR mixtures were treated with the KLD enzyme mix and transformed into NEB 5α competent cells. Colonies were selected on LB + AMP (100 μg/mL) plates, and plasmid DNA was harvested from single colonies using QiaQuick Miniprep Kits. Successful mutants were identified by Sanger sequencing (Eurofins Genomics), and approximately equal expression relative to Dd2 PfCRT was confirmed by Western blotting (see Methods). The *S. cerevisiae* strain CH1305 (MAT**a** *ade2 ade3 ura3–52 leu2 lys2–801*) was transformed with purified PfCRT plasmids via the LiAcetate method as done previously.^5^

**Table 1:** Probability of L7 Disulfide Bond Formation for Previously Published Energy-Minimized PfCRT Isoform Structures,^28^ as Predicted by SSBondPredict^29^

	probability of S–S formation	
energy-minimized PfCRT Structure^28^	for C301/C309	for C289/C312
HB3 AFMD	99.1%	88.2%
HB3 EMMD	99.2%	91.1%
Dd2 AFMD	99.6%	89.9%
Dd2 EMMD	98.8%	96.3%
7G8 AFMD	99.3%	89.6%
7G8 EMMD	99.7%	88.7%

### Yeast Growth Rate Analysis and CQ Transport Quantification

Live yeast cell PfCRT CQ transport assays were conducted as previously described.^6,30,33,34^ In brief, three independent colonies of each strain were grown in noninducing media [SD-URA: 3% glucose, 0.67% yeast nitrogen base without amino acids (YNB – AA), and 0.077% complete synthetic media without uracil (CSM – URA)], washed 3× with sterile water, and then seeded at OD_600_ = 0.1 per well in a sterile flat bottom 96-well plate with either PfCRT-inducing (SGR-URA: 3% galactose, 0.67% YNB – AA, 0.77% CSM – URA) or noninducing media (SD-URA), both with 100 mM HEPES, pH 6.75 and 16 mM CQ. Growth under each condition was then measured in duplicate or better for each colony (6–9 determinations in total). PfCRT and CQ-dependent control growth delays were calculated from the difference in time to reach the maximum growth rate under standard conditions (100 mM HEPES, pH 6.75, 16 mM CQ) for inducing (+PfCRT) vs noninducing (−PfCRT) conditions as described in detail elsewhere.^6,33,34^

### Live Cell Redox Buffered Growth Rate Analysis

Two redox buffer systems were developed to fix the redox potential during transport assays. As glutathione (GSH) has been proposed as a substrate of PfCRT,^35–38^ we avoided it and instead used nontoxic (Figure S1) oxidized and reduced forms of β-mercaptoethanol (ßME) and dithiothreitol (DTT) to span potentials of −40 to −320 mV. Redox buffered yeast growth rate plates were prepared as described above with some modifications. In brief, all wells of the plate included 1.5 mM of the reductant equivalents (reduced + oxidized forms, see SI Calculation of Redox Buffer Conditions), 100 mM HEPES, pH 7 SGR-URA, and 16 mM CQ (with midpoint potential values determined at pH 7 for ßME and DTT^39,40^ used, see the SI). Desired concentrations of oxidized and reduced ßME and DTT (Tables S2 and S3, respectively) were prepared from 10× stock solutions stored at −80 °C for no more than 2 weeks. CH1305 yeast/empty pYES2 (EV) was included as the negative control, and time to reach maximum growth rate for EV was subtracted from the time to reach maximum growth rate for the Dd2 PfCRT expressing strain at each potential. All data from 6 independent experiments were averaged and fit to a 4-parameter sigmoid. Midpoint potential shown is the average ± 95% confidence interval (see Results).

### DTNB Quantification of Free and Total Sulfhydryl Concentrations

To test stability of the redox buffers, a modified Ellman’s assay^41^ was used (Figure S2), allowing for quantification of free and total sulfhydryl concentration. 10 mM DTNB was prepared in 1 M Tris-Cl pH 8.2 (stored at −20 °C until use), and a standard curve consisting of serial dilutions of ßME from 3 mM to 2.93 μM were prepared in the same buffer. Redox-buffered yeast samples were grown as described above in 96-well plates, the supernatant was harvested via centrifugation, and 160, 170, or 180 μL of 1 M Tris-Cl was added followed by 10 μL of 10 mM DTNB solution to 20 μL of either standard or sample. The plate was allowed to incubate in the dark for 15 min at RT prior to measuring the absorbance at 412 nm. To determine total sulfhydryl concentration, chemical reduction by sodium borohydride was first done prior to Ellman’s assay (see Figure S2A vs S2B).

### Molecular Dynamics

Protein preparation and our MD procedure for PfCRT (see https://www.schrodinger.com/platform/products/desmond/) were as in ref. 28, with minor modifications. The Dd2 EMMD structure was used as a starting point for all *in silico* mutagenesis and MD simulations. Maestro’s ‘Residue and Loop Mutation’ tool was used to generate the reduced form of Dd2 PCRT *in silico*. Structures were generated from 3 × 10 ns or 3 × 1 μs randomized starting velocity simulations.^28^ Assigning L7 C with broken (free thiol, reduced) or fixed disulfides (oxidized) was during protein preparation prior to imbedding PfCRT within a POPC bilayer and subsequent MD. Simulations were done using Desmond as described elsewhere.^28^ Resulting ‘.trj’ files were merged via the Linux script ‘out.cms’ file before they were reimported into Maestro. The trajectory frame clustering tool in Maestro was used to compute protein structure once the simulation converged^28^ (Chain A, frequency = 1, 1 cluster), and the cluster file was then reimported into Maestro, where lipids and waters were removed for ease of visual comparison. Resulting average protein structure.pdb files were imported into UCSF ChimeraX where proteins were aligned with the Matchmaker tool using the best chain alignment algorithm. The root-mean-square deviation (RMSD) tool was used to calculate the RMSD values. Each calculation began with a default system relaxation protocol as in ref 23 followed by simulation in an isothermal, isobaric NPT ensemble with constant particle number (*N*), pressure (*P*; 1.01325 bar), and temperature (*T*; 310 K).

### Small-Scale Crude Membrane Isolation

The crude membrane (CM) isolation procedure was as in ref 5. In brief, the culture was grown at 30 °C to OD_600_ = 1, and cells were pelleted, washed twice with sterile water, and resuspended in 30 mL of inducing SGR-URA media. Cells were grown for another 24 h at 30 °C and OD recorded. Cultures were pelleted, resuspended in 1 mL of harvest buffer (0.1 M glucose, 50 mM imidazole, 5 mM ßME, pH 7.5), and transferred to 1.7 mL Eppendorf tubes. Cells were pelleted and washed with 1 mL of harvest buffer × 2, and 600 μL of glass beads (0.5 mm) previously washed with H_2_O ∼10 times and then breaking buffer (250 mM sucrose, 100 mM glucose, 50 mM imidazole, 1 mM MgCl_2_, Pierce EDTA-free protease inhibitor cocktail (PIC) [ThermoFisher, CAT: A32965] (1 tablet/10 mL), 5 mM ßME, pH 7.5) × 3 were added. The suspension was incubated on ice × 5–10 min and then vortexed to lyse cells ([vortex for 30 s followed by 30 s on ice] × 30). Glass beads were pelleted, and the supernatant was transferred to a fresh Eppendorf tube and centrifuged at 2500*g* × 5 min. The resulting supernatant was transferred to an air-ultracentrifuge tube and centrifuged at 15,000 rpm for 20 min in a benchtop air-ultracentrifuge, and the supernatant, containing cytosolic proteins, was collected and stored at −80 °C. Membrane pellets were resuspended in suspension buffer (1 mM MgCl_2_,10 mM imidazole, EDTA-free PIC (1 tablet/10 mL), pH 7.5) and stored at −80 °C for protein quantification and Western blot analysis (see below). Larger-scale membrane preparations were prepared similarly, except cells were lysed using a microfluidizer instead of a glass bead vortex.

### Modified Amido Black Protein Assay

To quantify protein in harvested yeast fractions, we utilized a modified Amido Black assay adapted from ref 42. Samples and BSA standards were aliquoted into a 96 well plate. Protein was solubilized and denatured in 1% SDS, 1 mM Tris (pH 7.5) at 37 °C for 10 min, precipitated with 30 μL of 50% TCA, and incubated for 15 min at RT. Each sample was then collected using a prewetted 0.45 μm mixed cellulose ester MultiScreen filter plate under vacuum, and each well was rinsed with 100 μL of 6% TCA and stained with 100 μL of Amido Black staining solution (0.1% Amido Black, 45:10:45 45:10:4MeOH:HOAc:H_2_O) for 6 min. Staining solution was removed via inversion and repeated tapping onto a paper towel, filters were rinsed with distilled water for 30 s, destained with 100 μL of destaining solution (90:2:8 MeOH:HOAc:H_2_O) for 2 min, and rinsed with distilled water again, and the immobilized, stained protein was eluted into 100 μL of spot remover (25 mM NaOH/50 μM Na_2_EDTA, 50% ethanol) with gentle shaking (950 rpm, Eppendorf Mix Mate) until no stained protein remained on the filters. Solutions were transferred to a clear bottom 96-well plate, and absorbance at 630 nm was recorded. Internal BSA standards were used to construct a standard curve, and the protein concentration of samples was extrapolated.

### Ni-His Affinity Chromatography Pull Down and Elution

Yeast CM were added to solubilization buffer (0.5% w/v dodecyl-β-maltoside (DDM) in 2× wash buffer [100 mM phosphate or 100 mM Tris with 1 M NaCl, pH 7.5, 500 mM sucrose, 2 mM MgCl_2_, 40% glycerol]) and mixed for 1 h with gentle revolving inversion at 4 °C, and 500 μL of Ni-charged nitrilotriacetic acid (Ni-NTA) resin (Bio-Rad, cat: 7800800) in storage buffer (20% EtOH) was then added (∼250 uLs of beads). Ni-NTA beads were prepared by washing 3 times with solubilization buffer. Detergent-extracted membrane solutions were added to Ni-NTA beads, tubes were mixed with gentle revolving inversion at 4 °C for 2 h, Ni-NTA beads were pelleted, and the supernatant was saved. Beads were washed with 1 mL of column wash buffer (0.05% v/w *E. coli* lipid, 20 mM imidazole, 6.5 mM ßME, EDTA-free PIC in 1× wash buffer) for 5 min by inversion and pelleted, and the supernatant was collected. Finally, 1 mL of elution buffer (1 M imidazole, 6.5 mM ßME, EDTA-free PIC in solubilization buffer) was added to the resin, the tubes were mixed by inversion at 4 °C for 15 min, beads were pelleted, and the supernatant was collected for Western blot analysis.

### Biotin Maleimide Labeling

To explore the equilibrium between SH HS and S–S for cysteine pairs, we utilized covalent maleimide labeling of free SH. Purified membranes ± PfCRT were resuspended at pH 7.0, and [maleimide] (M) or [maleimide-biotin] (MB) (Figure S4) was added to a final concentration 13–15 fold higher than the protein concentration. One assay format (″exp’t A″ [Figure S4, Table S5]) reacts heterologously expressed PfCRT with MB in the presence of excess reducing agent (to label all PfCRT C), then solubilizes the membrane with 0.1% dodecyl maltoside (DDM), to isolate MB labeled PfCRT using the C-terminus dodecaHis tag and Ni-chelating beads (above), and resolves MB-labeled PfCRT using SDS/PAGE followed by Western blot detection with an avidin–HRP antibody and band quantification via densitometry. The second (″exp’t B” [Figure S4, Table S4]) first blocks all free cysteine SH under ambient conditions with nonbiotinylated maleimide, then fully reduces any previously existing PfCRT S–S not labeled with M, reacts those liberated thiols with MB, and quantifies labeling as in ″exp’t A″. Side reactions of M or MB with thiol containing reducing agents were avoided by increasing M/MB concentrations for those reactions and incubating membranes in buffer for 2 h prior to M/MB addition. Non-thiol containing TCEP was used to buffer redox potential in confirmatory labeling experiments (c.f. Figure 5, Table S4).

## Results

Using the PfCRT atomic resolution structure, we observed by inspection that only two C pairs within L7 (the 4 C within loop 7 ([L7] connecting helices 7 and 8), out of 14 total C (Figure 1), reside close enough to theoretically form S–S disulfide bonds (∼2.05 Å).^1,28,29,43^ These are the C289/C312 and C301/C309 pairs, with S to S distances of 2.02 and 2.04 Å, respectively.^1,28^ These four C are absolutely conserved across all 24 known CRT proteins found in other *Plasmodia* spp., in contrast to the remaining 10/14 C (Figure 1).

**Figure 1:**
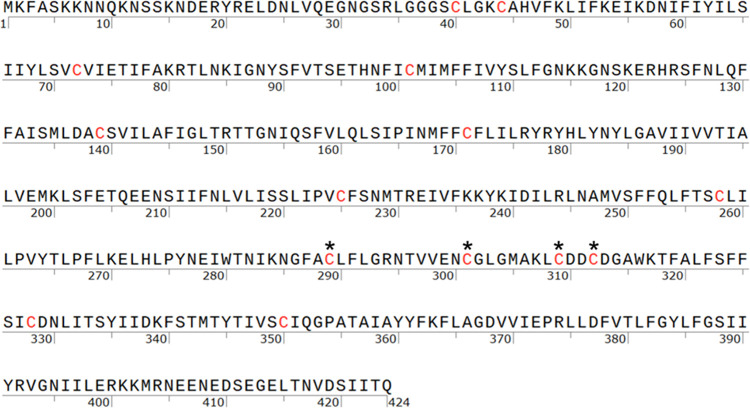
Location of cysteines in the Dd2 PfCRT primary sequence. The primary sequence of the CQR-conferring Dd2 PfCRT isoform contains 14 C residues (marked in red) distributed throughout the protein. The loop 7 residues (C289, C301, C309, & C312) are labeled with *. All 14 C are conserved in the chloroquine-sensitive (CQS) associated “HB3” wild-type PfCRT isoform (not shown; see elsewhere^28^).

Four of the other 10 C are conserved in 23/24 of known CRT orthologues (C101, C171, C328, and C350).^28^ The 4 L7 C are disposed within the highly redox active DV (Figure 2).

**Figure 2:**
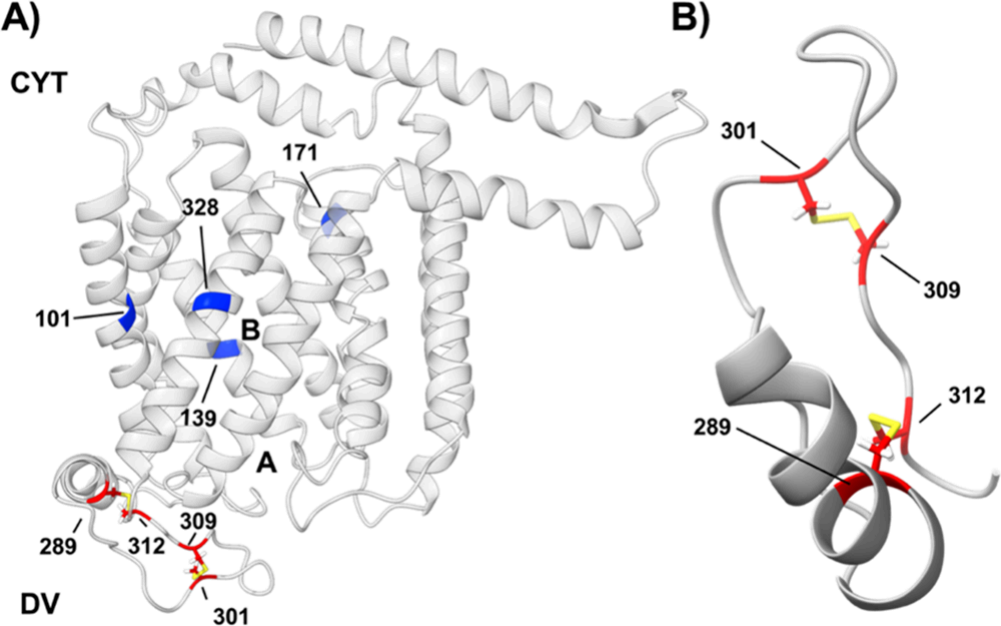
Distribution of C residues within the PfCRT tertiary structure. (A) The Dd2 AFMD structure^28^ of PfCRT with L7 C residues (red, bottom) and 4 other C that, as described below, when substituted to S or A yield compromised but still active Dd2 PfCRT (dark blue). DV and CYT indicate the DV and cytosolically disposed sides of the membrane containing PfCRT, respectively. (B) Closeup view of the JM2 region (residues 279–292) and the ‘lariat’ segment of L7 (residues 293–313) showing predicted disulfide bonds between C289/C312 & C301/C309 (yellow).

Other C are well conserved, including C72 (found in 22/24 orthologues), C258 (18/24), and C139 (13/24) with CRTs having either S (positions 72, 139) or L (258) at the corresponding codons.^28^ These would require a single (to encode S) or double (L) base substitution in *pfcrt.* Substitution to L found in rodent malaria spp. might arise from a combination of mutational and translational selection, since rodent *Plasmodia* encode a smaller tRNA repertoire relative to the human spp.^44^

We used SSBondPredict^29^ software to assess the likelihood of disulfide bond formation and found that only C301/C309 and C289/C312 were likely to form S–S within PfCRT (Table 1). These were found for PfCRT isoforms expressed in either CQS (HB3) or CQR (e.g., 7G8, Dd2) cognate strains of *P. falciparum*. No others were predicted at probability >50% for any PfCRT isoforms examined.

S–S putatively formed from the 4 conserved L7 C disposed within the DV (Figure 2) are expected to respond to oxidative perturbations since the DV is the site of redox active heme release during Hb catabolism that generates an ROS cascade, as well as high [GSH] that acts to neutralize the ROS repercussions of this cascade.^10^ L7 is in precisely the correct location to respond to changes in DV redox potential caused by introduction of antimalarial drugs^10^ that perturb detoxification of Hb heme to hemozoin.^45,46^

We mutagenized all 4 individual L7 C to S or A to create single C mutants, C289S/A, C301S/A, C309S, and C312S, and created C289/C312 to S or A and C301/C309 to S or A “double C” mutants, as well as L7 C quadruple mutants (C289/C301/C309/C312, L7S/A 4, all C to either S or A) (see Table S8). To assay mutant function, we used previously defined CQ and PfCRT-dependent growth inhibition of yeast expressing different PfCRT isoforms as a quantitative measure of relative PfCRT isoform CQ transport (Figure 3 and cf. Table S7).

**Figure 3:**
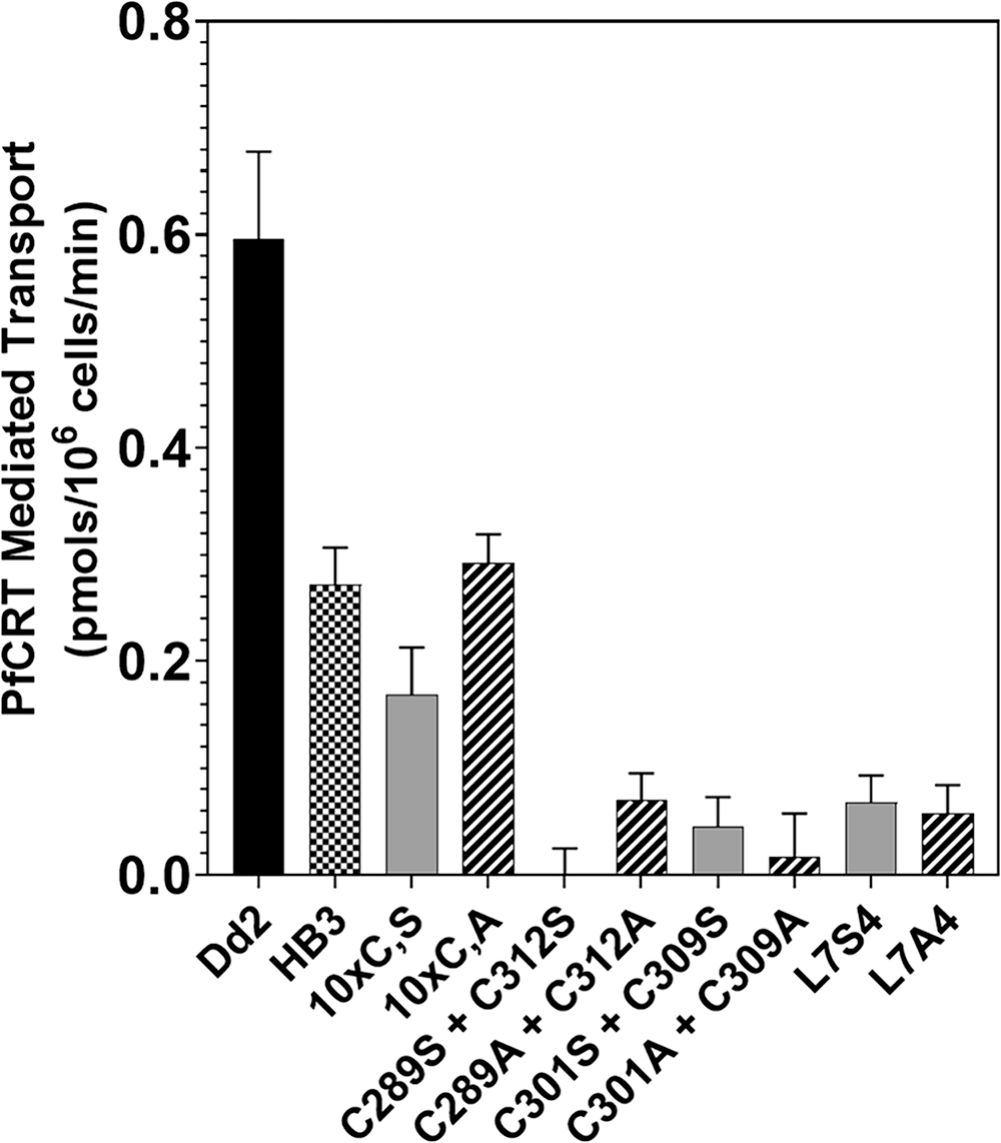
Disruption of putative L7 disulfide bonds by substitution of C with S or A abrogates the CQ transport ability of CQR PfCRT, yet no other C is obligate for PfCRT-mediated CQ transport. Average (*n* = 3) PfCRT-mediated CQ transport^5,34^ under standard conditions (see methods) for CH1305 yeast expressing Dd2 (left, black bar), HB3 PfCRT (checkered), mutant PfCRT with all non-L7 cysteines substituted with S (10×C,S) or A (10×C,A) (gray or striped), or Dd2 PfCRTs with double (C289S/A + C312S/A, C301S/A + C309S/A), or quadruple (C289S/A + C312S/A + C301S/A + C309S/A; ″L7S/A4″ [right]); see also Table S7.

We found that, within the resolution of our method,^34^ any of the individual L7 C to S or A mutants showed a complete loss of PfCRT-mediated CQ transport function relative to Dd2 PfCRT harboring all 14 C (Table S7) and that simultaneous substitution of all 4C within loop 7 with either S or A (″L7S/A4″) also resulted in inactive PfCRT (Figure 3, right, Table S7). The same loss of function upon simultaneous substitution of all 4 L7 C with either S or A was also observed when the mutations were created for the HB3 PfCRT isoform (Table S7). In contrast, none of the other 10 C within PfCRT proved obligate for function as “10 C to S or A” (10×C,S/A) PfCRT mutants harboring all possible 10 C to S or to A substitutions but including the 4 C found in L7 (C289, C301, C309, and C312), were able to transport CQ at ∼30% (10 S mutant) or ∼50% (10 A mutant) efficiency relative to Dd2 PfCRT, respectively. (Figure 3 and Table S7). We identified the highly conserved C C101, C139, and C171 as well as the universally conserved C328 as all having small contributions to CQ transport, as evidenced by some decrease in transport upon single C to S or A substitution at these positions (Table S7). For example, substitution of only C101 resulted in Dd2 PfCRT mutants with 60–70% transport activity relative to Dd2 PfCRT harboring all native 14 C (Table S7).

Using these mutants, we next tested which C can be efficiently labeled with the irreversible covalent free thiol label maleimide and whether labeling is dependent upon reducing vs oxidizing conditions. That is, if a C in question existed as a free thiol under ambient conditions, it would be available for covalent labeling with either maleimide (M) or maleimide–biotin (MB), but if the C residue thiol participated in forming a S–S bond with another thiol of a neighboring C, it would not be available for labeling until the protein was fully reduced to below that S–S bond equilibrium redox potential.

Thus, we labeled Dd2 PfCRT vs Dd2 PfCRT L7S4 and double L7C mutants with MB under fully reduced conditions to first label all available C (e.g., all 14 C for the Dd2 protein Figure 4, lane 1; Table S8), vs 10/14 residue thiols for the L7S4 Dd2 PfCRT mutant, (Figure 4, lane 2, Table S8), and vs 12/14 for the two L72C mutants (Figure 4 lanes 3 and 4, Table S8). To probe the L7 disulfide bond dynamics, C with free thiols in Dd2, L74S, etc. under ambient conditions, they were also first reacted with underivatized maleimide under ambient conditions prior to PfCRT reduction (″exp’t B″, c.f. Figure S4) to covalently block free SH with M, and then reacted with MB following complete reduction of PfCRT with TCEP, which allowed the subsequent labeling of any C thiol sequestered within a disulfide bond under ambient conditions. The results are clear and dramatic: Dd2 PfCRT reacted with MB prior to vs after blocking with M (″exp’t A″ vs ″exp’t B″ [Figure S4]) shows 14 vs 4 thiols labeled with MB (lane 1 vs lane 5 Figure 4; Table S8) whereas L7S4 mutant Dd2 shows 10 vs 0 (lanes 2 vs 6) and each of the two L72C mutant shows 12 vs 2 (lanes 3 vs 7 and 4 vs 8; see also Table S8), with the C301/C309 double mutant slightly less well labeled (lane 8 vs 7 bottom) suggesting that C289/C312 may be slightly easier to reduce, although the result is not statistically significant (Table S8). In contrast, 10×C,S PfCRT shows 4 C labeled in both experiment A and B formats (Figure S5, lanes 5 vs 9).

**Figure 4:**
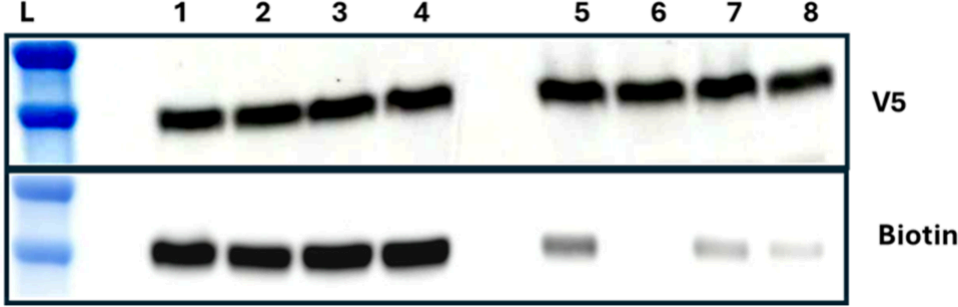
MB labeling clearly shows the presence of individual disulfide bonds for L7 C 289/312 and 301/309 pairs. V5 tag detection (top) and biotin detection (bottom) of PfCRT in 12×His-nickelbead pulldowns of PfCRT from maleimide reacted yeast membranes harboring equal levels of Dd2 PfCRT (lanes 1 and 5), mutant Dd2 L7S4 (lanes 2 and 6), mutant Dd2 C289S/C312S (lanes 3 and 7), and mutant Dd2 C301S/C309S (lanes 4 and 8). Bottom left (lanes 1–4) are maleimide biotin (MB)-only reaction conditions (left), whereas bottom right (lanes 5–8) are maleimide (M) blocking followed by MB (right) conditions, described earlier as “exp’t A”, lanes 1–4, or “exp’t B”, lanes 5–8 (see Figure SI4). Labeling ratios (shown in Table S8) were calculated by dividing the V5-normalized biotin band intensity by that of the MB (biotin) band (see caption to Table S8).

That is, quantitative densitometry of the biotinylated band intensity divided by the V5 band intensity that quantifies the total amount of PfCRT in the same sample shows the binding of MB to free C thiols in PfCRT present under different conditions (Figure 4). We routinely used the same amount of membrane-imbedded PfCRT for each labeling reaction, as evidenced by equal V5 band intensities across all lanes (Figure 4 top) and labeled at least 3 independently purified PfCRT-containing membrane preparations under each condition to calculate the number of labeled C ± SD under each condition for each mutant (see Table S8 caption). Interestingly, given the labeling intensity found for all 14 C (Figure 4, lane 1 Table S8), intensity is nearly identical to what is expected for 4/14 C under experiment B conditions (3.92, lane 5; c.f. Table S8), in which the SH of the free non-L7 10 C are first “blocked” by reacting with nonbiotinylated maleimide (M) under ambient conditions, and the protein then fully reduced with TCEP and then reacted with MB to reveal SH that were previously sequestered as S–S (Figure 4, lane 5). This is as predicted (as is also suggested by MD^28^) if the 4 L7 C form S–S under ambient conditions and are thus unavailable for maleimide labeling until the protein is fully reduced. The other 10/14 C not within L7 (Figure 1 and 2) are in the form of free thiols under ambient conditions and do not form disulfide bonds; therefore, they readily react with M under ambient conditions and cannot then react with MB when PfCRT is reduced. Labeling of the L7S4 (below) and 10×CS (Figure S5) Dd2 PfCRT mutants test and verify this conclusion.

As shown, the L7S4 mutant (only the four L7 C substituted with S) yields 10/14 labeling intensity via experiment A (Figure 4, lane 2 and Table S8) but 0/14 labeling of the same protein during experiment B (Figure 4 lane 6), whereas labeling of the 10×C,S Dd2 PfCRT shows 4 C labeled under both exp’t A and B (Figure S5 lanes 5, 9). Taken together, these data clearly show that the 4 L7 C form disulfide bonds under ambient conditions and suggest that PfCRT CQ transport activity may be influenced by SH/HS to S–S interconversion involving both pairs of these C.

We next tested whether the two redox active thiol pairs could be labeled individually. When both C289 and C312 are mutated to S in the Dd2 protein, 12/14 MB labeling intensity is found under “exp’t A” conditions (Figure 4, lane 3, Table S8) and 2/14 MB labeling intensity is found under “exp’t B” conditions (Figure 4 lane 7, Table S8), as is also the case for the double C301S/C309S mutant (Figure 4 lanes 4 and 8, Table S8). Taken together, these data show that residues C289/C312 and C301/C309 found within L7 form the only disulfide bonds present within PfCRT, that the two S–S bonds form independently, and that loss of any 1 disulfide results in abrogated CQ transport activity (Figure 3).

### Redox Titration of Dd2 PfCRT Function and Labeling

To test for any correspondence between L7 C S–S interconversion and CQ transport function of PfCRT, we devised redox buffering conditions that spanned −0.04 to −0.4 V (SHE) using 2-mercaptoethanol [ßME] monomer/dimer, dithiothreitol [DTT]/cyclic DTT [cDTT], or tris­(2-carboxyethyl)­phosphine [TCEP] and TCEP oxide [TCEPO] redox partners (see SI, Tables S1–S3). With redox potential buffered at these values and using redox buffered PfCRT transport assays vs CQ perfected earlier,^5,6,30,34^ we find that Dd2 PfCRT is 100% active when fully oxidized, but essentially inactive when all L7 C are free SH (Figure 5). We find that Dd2 PfCRT is >50% active at potentials > −140.5 ± 14.4 mV or > −119.1 ± 7.2 mV (Figure 5) when the potential plotted (*x* axis) is that calculated at the beginning of the experiment (solid line, closed circles) or directly measured by Ellman’s assay (dashed line, open squares) after 2 days of yeast growth, respectively.

**Figure 5:**
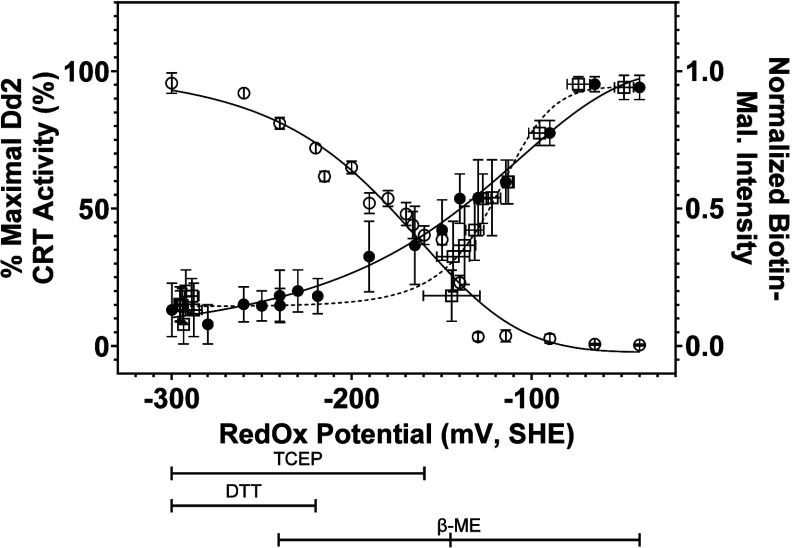
Maximal PfCRT function occurs upon formation of L7 disulfide bonds. Percent maximal Dd2 PfCRT activity (left axis) measured under redox potential-buffered growth curve assay conditions (*n* = 6) plotted against the initial set redox potential (black circles, solid line) or against redox potential directly measured after 2 days of yeast growth (open squares, dashed line). Also plotted is V5 normalized MB labeling intensity of the 4 L7 C in Dd2 PfCRT under redox potential-buffered conditions (open circles, solid line, right axis) [*n* = 3; from 3 independent labeling experiments and 3 independent Western blot analyses] indicating SH HS/S–S interconversion. For the ″modified exp.’t B″ labeling measurements, Dd2 PfCRT was first blocked with M, then redox buffer was added and MB labeling was done without adding excess TCEP reducing agents. The results show that maximum CQ transport correlates with maximum L7 S–S bond formation. Brackets underneath the *X*-axis indicate the redox buffer system used to achieve potentials set during functional analysis or labeling (ßME/ßME-Dimer −40 to −250 mV, DTT/cDTT −215 to −350 mV, and TCEP/TCEPO −150 to −340 mV; see Tables S1–S3). The TCEP buffer system was used for maleimide labeling since it lacks free thiol, whereas the DTT buffer system was used for the redox-buffered TeCan PfCRT function experiments, and the ßME buffer system was used for both (ßME vs TCEP buffering to the same potential yielded the same PfCRT activity results; Figure 3 left). Solid lines represent the best fit for a four-parameter sigmoid, and error bars represent SEM. The midpoint potentials were found to be −178 ± 11.9, −140.5 ± 14.4, and −119.1 ± 7.2, mV (average ± 95% C.I.) for PfCRT 4 L7 C labeling, PfCRT function vs initial set redox potential, and PfCRT function vs measured redox potential after 2 days of yeast growth, respectively.

Not coincidentally, L7 C are <20% labeled at potentials > −140 yet are fully labeled at potential < −250 (more negative potential) where PfCRT does not transport CQ (Figures 5 and 6, Table S8), showing that both C289/C312 S–S and C301/C309 S–S bonds are required for full PfCRT CQ transport function.

**Figure 6:**
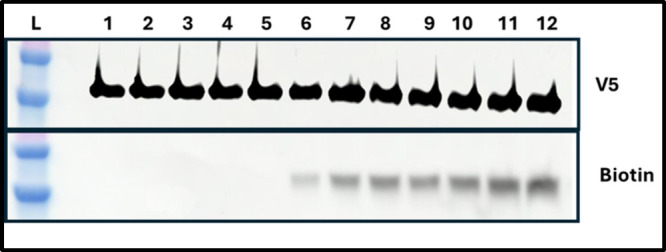
Labeling of L7 disulfides as a function of redox potential. V5 detection (top) and biotin detection (bottom) of 12×His-nickel bead pulldowns of Dd2 harboring CMs buffered using ßME/ßME-dimer redox buffers (*x* axis). Lanes 1–11 show Dd2 under experiment B conditions with decreasing potential as described in Table S2 (−39.98, −64.86, −89.89, −114.38, −129.67, −139.81, −149.70, −164.86, −190.28, −215.05, and −239.95 mV, respectively). Lane 12 is the result for Dd2 experiment B under ambient conditions as an internal control, showing 4C labeling (Table S9). Labeling ratios (shown in Table S9) were calculated from the ratio of the V5-normalized biotin band intensity to that of the band for Dd2 (set to 4 labeled C, lane 12, Table S9).

Taken together, these data indicate that C289/C312 S–S can form in the absence of C301/C309 S–S, and vice versa, and that both are important for PfCRT function. PfCRT missing either or both disulfide pairs is virtually inactive (Figures 3–5). There must therefore be a catalytically active conformation of PfCRT in the presence of both S−S bonds that is not present when both are missing.

### Computational Analysis of Oxidized and Reduced Dd2 PfCRT

To investigate this possibility, we performed MD calculations as performed previously^28^ to test how disulfide–sulfhydryl interconversion at the C289/C312 and C301/C309 pairs might affect L7 conformation and overall PfCRT structure. Figure 7A,B shows the C_α_ RMSD for the Dd2 EMMD^28^ structure modeled with all 4 L7 C fixed as free thiols ([reduced] colored, opaque) vs with L7 C289/C312 and C301/C309 disulfide pairs ([oxidized] gray, transparent). Views shown are for the entire protein defined by cryo EM^1^ (A) or the expanded L7 + JM2 region (B). Also shown in each panel is the highest affinity energy minimized docked CQ^2+^ pose (green) for drug binding site “A”.^28^ These data illustrate a significant deviation >5 Å that occurs between the ″tip″ of the L7 lariat and the DV disposed ends of pore-forming helices near putative drug binding site A^28,47^ upon free sulfhydryl–disulfide interconversions involving the four L7 C, regardless whether MD trajectories are 10 ns or 1 μs in duration (Figure 7C; Figure S10).

**Figure 7:**
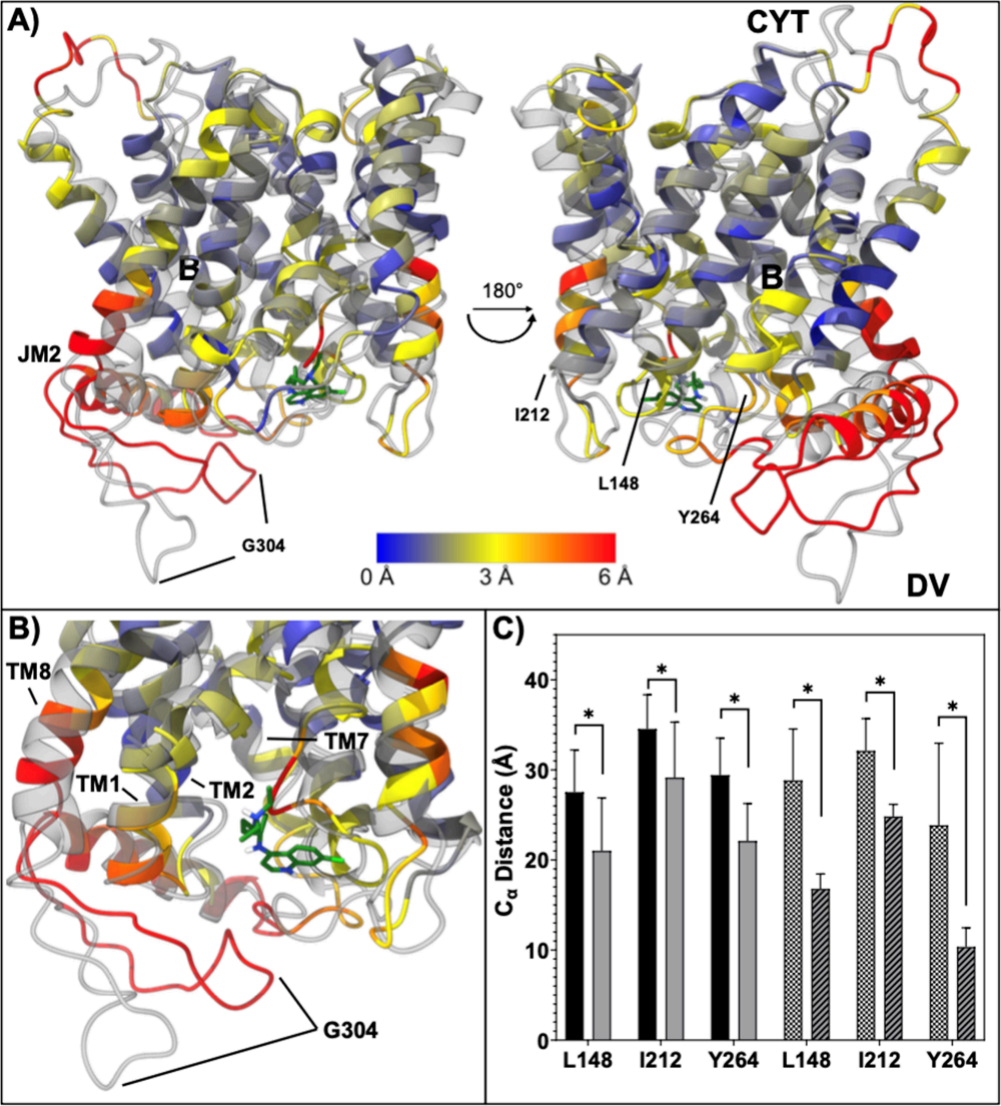
Loss of L7 disulfides leads to conformational changes in L7. (A) Deviations in the average structures of fully reduced Dd2 EMMD ([L74C = SH] colored, opaque) vs oxidized Dd2 EMMD ([L74C = S–S] gray, transparent) denoted by a sliding color scale. The highest affinity pose of chloroquine [CQ^2+^; green], as described in^28^ is shown in Site A. The putative location of drug binding site B^28^ is denoted with “**B**”. Incomplete JM helices 0, 1, and 3 (residues M1 – N58 & I396 - N405) not evident in the original cryo-EM structure^1^ were omitted for clarity. The reduced and oxidized structures were aligned using ChimeraX’s Matchmaker tool using the best-chain alignment algorithm and colored by C_α_ RMSD, with deep blue being 0 Å, yellow being 3 Å, and red being ≥6 Å. The minimum, maximum, and average C_α_ RMSD across the entire protein were 0.24, 11.2, and 2.7 Å, respectively. Residues used for quantifying changes in position of the L7 ‘lariat’ segment (e.g., G304 at the terminus ″tip″ of the L7 lariat, L148 at the DV end of TM3, I212 at the DV end of TM6, and Y264 at the DV end of TM7). These three residues were chosen since they are circularly disposed like a ring around the DV opening of CQ^2+^ binding site A. Relative distance between these residues plateaus smoothly during the MD simulation (see Figure S11). (B) Close up view of structural changes in L7 for the oxidized (gray, transparent) vs reduced (colored, opaque) structures. We note three L7 segments. These consist of the initial strand following TM7 and preceding JM2, the JM2 helical motif, and the L7 ‘lariat’ strand, which follows JM2 and precedes TM8. Upon reduction and loss of disulfide bonds, we envision that the L7 ‘lariat’ shows an upward movement toward the DV disposed ends of TMs 2, 3, 6, and 7 into the mouth of the pore near drug binding site “A” (shown with CQ^2+^ [green] docked as described; note the position of G304 at the tip of the lariat for reduced [in color] vs oxidized [gray] structures). A concomitant small backward movement of the L7 ‘lariat’ toward the base of TM7 is also seen in our MD simulations. L7 also contains E271, a residue previously identified as being involved in CQ^2+^ binding to Site A in Dd2 PfCRT.^28,47^ (C) Cα distance between residue G304 (the tip of the L7 ‘lariat’) and residues L148, I212, and Y264 computed for 3 × 10 ns (left 6 bar) and 3 × 1 μs (right 6 bar) MD simulations of oxidized vs reduced Dd2 PfCRT (see also Figure S10). Oxidized 10 ns is shown in black vs reduced 10 ns shown in gray and oxidized 1 μs is shown as checkered vs reduced 1 μs shown as striped. Error bars correspond to SD. * indicates a significant *p*-value (<0.01) by Welch’s unpaired *t* test. The residues used here for distance calculations are also shown in expanded Figure S6 for clarity, and the fluctuation in avg distances (″noise″ in the plot vs simulation time) that occurs during a simulation can be seen in Figure S11.

That is, when PfCRT is reduced, the ‘lariat’ portion of L7 (Figure 7, Figure S6) shows an upward movement toward the base of TMs 1, 2, and 7 that define the putative drug-binding site A^28,47^ for CQ (docked CQ shown in green) and a slight backward movement closer to TMs 1 and 2 regardless whether MD trajectories are 10 ns or 1 μs. The “fulcrum” for each of these movements begins at the C301/C309 pair, indicating that when oxidized, the C301/C309 S–S restricts the loop from large movements. This region also contains residue E271, which has previously been identified as likely involved in CQ^2+^ binding to Site A.^28^ Not coincidentally, the lariat segment shows a large redox-dependent deviation of >6 Å, moving downward and forward toward the rest of the pore opening when oxidized. The upward and backward movements of the ‘lariat’ were quantified (Figure 7C) via calculating the average C_α_ distances between G304 at the tip of the L7 ‘lariat’ and residues L148 at the base of TM2, I212 near the base of TM6, and Y264 at the base of TM7. Over the course of all simulations, we observed a statistically significant decrease (*p* < 0.01 by Welch’s unpaired *t* test) in these distances of ≥6 Å for the reduced vs the oxidized forms regardless whether calculated energy minimization trajectories are 10 ns or 1 μs (Figure S11). In contrast, we observed *a* < 2 Å movement for residue E207 (Figure S7), which was previously identified as a key residue in the pH dependence of PfCRT mediated CQ transport.^48^ This residue was proposed to form a transient salt bridge with K80 ^48^; however, this was based on the residues’ distance approximated from a homology modeled structure, not calculated via MD (Figure S8).

Electrostatic profiling of the mouth of the DV disposed pore for the cryo-EM PfCRT structure (Figure 8) reveals significant charge density (red = negative, blue = positive) within drug binding site A, and this profile also changes upon L7 sulfhydryl/disulfide interconversion (Figure 8A vs B). Negative charge is due to 4 aspartic acid residues D310, D311, D313, and D368 and glutamic acids E299, E204, and E198 that do not directly interact with CQ^2,28^ as well as E271, E207, and key site A^28^ residue N84 that are predicted to interact directly with drug^28^ (Figure 8A). The electron density and/or position relative to docked CQ of all of these side chains changes in oxidized (A, top) vs reduced (B, bottom) conformations (note, E271 and D137 are not visible by eye in Figure 8 since they are shielded in this view by other residues; e.g., K270 shields view of E271). Most dramatically, however, the G304 segment within the lariat is seen to sterically block DV access to drug binding site A in reduced (B, bottom) vs oxidized PfCRT (A, top; Figure 8).

**Figure 8:**
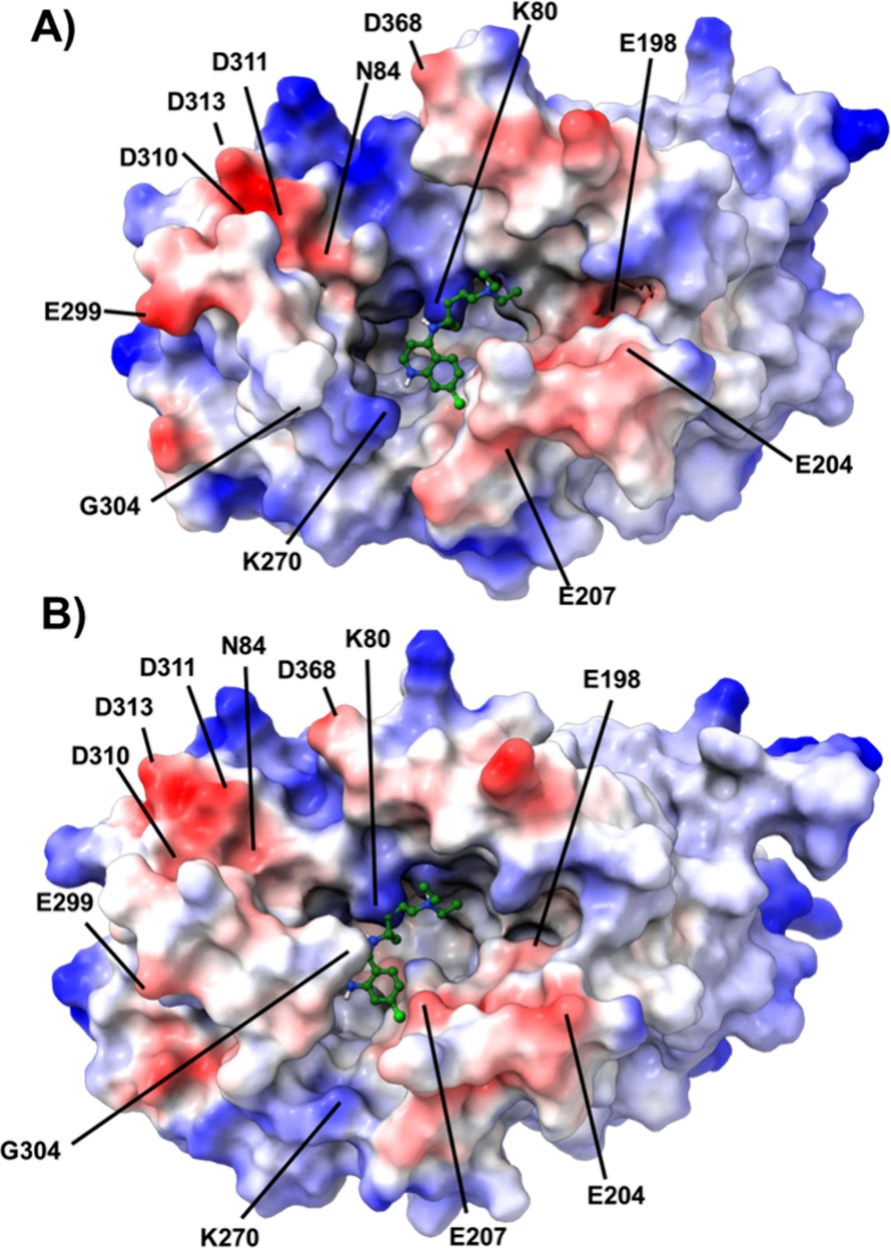
Changes in the DV disposed side of PfCRT upon reduction of L7 C. Calculated electrostatic potential (ESP) surfaces for the average minimized (A) oxidized and (B) reduced Dd2 pore opening oriented toward the DV interior. Residues contributing significant charge are annotated. ESP surfaces were calculated and displayed using ChimeraX, with red corresponding to negative charge, white to neutral, and blue to positive charge. The highest affinity pose of CQ (green), as described elsewhere,^28^ is shown in drug binding site A.

The negative charge near site A likely acts as an attractive force, guiding the drug to dock in site A where CQ^2+^ is then further stabilized by surrounding residues as described earlier^28^ (Figure 8A). Upon reduction of L7 C, the pore is partially blocked by the G304 lariat region. The redox-dependent change in pore access sterically hinders drug association with site A and likely interferes with the electrostatic attraction of positively charged CQ^2+^.

Not coincidentally then, we also observed significant changes in some side chain–side chain hydrogen bond (HB) and salt bridge (SB) lifetimes between the reduced SH vs oxidized S–S simulations for Dd2 PfCRT (Table 2). Specifically, the HBs between N84/D368, Y109/R244, and E232/T342 as well as others appear to be longer lived for the oxidized form, whereas the K85/D311 SB and T82/T318 and Q253/T256 HB appear to be longer-lived for the reduced form (Table 2). In contrast, the previously noted very stable (∼95%) lifetime^28^ E198–N154 HB appears to remain intact in both; hence, E198 is not found within Table 2.

**Table 2:**
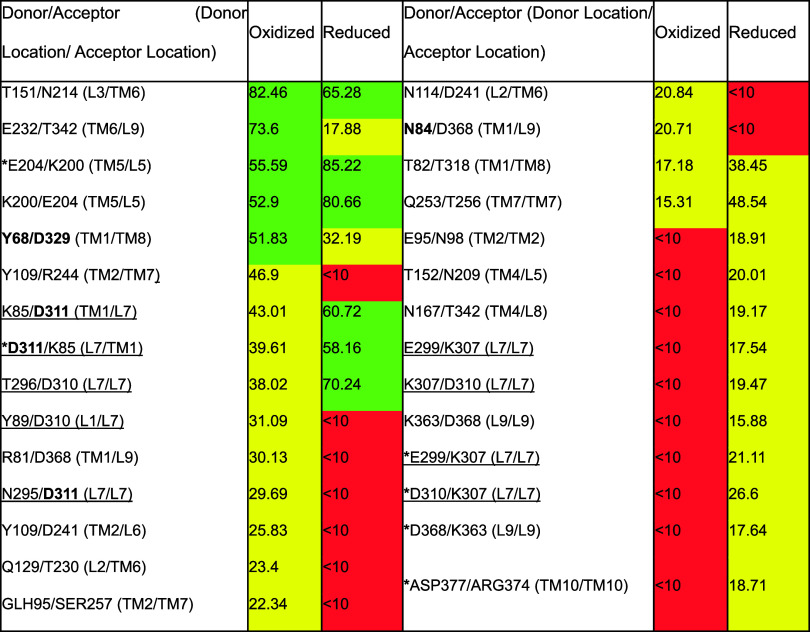
Different Lifetimes for Salt Bridges (SB; Asterisk; ≤ 4 Å Heteroatom to Heteroatom) and Hydrogen Bonds (HB; ≤ 3.2 Å Heteroatom to Heteroatom) That Appear for ≥ 10% of MD Calculation Time for the Oxidized vs Reduced Dd2 PfCRT EMMD Structures^a^

a Interactions involving a residue found within L7 are underlined, and interactions are listed in the order of lifetime in the oxidized Dd2 PfCRT EMMD energy-minimized structure. Residue pairs are listed as “donor”/”acceptor” with parentheses indicating the location of the residues within the protein. Bold residues indicate those identified in drug binding sites A or B. Color code denotes lifetime frequency (>50%, green; 10–50%, yellow; <10%, red).

Changes in SB or HB appear to be preferentially clustered near the pore openings of PfCRT (Figure 7), the pore-lining faces of TMs 2 and 7, and drug binding site B that involves these helical faces. For example, notably, site A residue Asn84 ^28^ appears to show clear changes in HB lifetime for the free SH (reduced) vs fixed S–S (oxidized) structures (Table 2). In the absence of S–S, D311 appears to preferentially H-bond to K85 rather than to N295, and multiple residues within the L7 ‘lariat’ also appear to show a significant change in HB lifetime as well as changes in their HB or SB partners upon reduction of all four L7 C, including N295, T296, K307, D310, and D311 (Table 2). Additional modeling and direct experimentation will test these hypotheses.

Since 2 of the 4 L7 C are found in a CXXC motif, which can be associated with formation of a divalent metal binding site, we investigated this possibility using the tertiary structure-based metal binding site prediction tools AlphaFold3 ^3^ and Metal3D.^2^ We note that the software returns merely predictions and that further direct experiments are needed to test these predictions. However, via these predictions, no L7 C was implicated by either program at a meaningful probability (e.g., > 20%) as belonging to a metal binding site, possibly due to the absence of additional side chain metal ligands nearby (Figure S9A,B).^49,50^ The metal binding sites that were instead predicted did not include Cs within the CXXC motif. That is, both AlphaFold3 and Metal3D did identify higher probability putative divalent metal binding sites within the pore of PfCRT (Figure S9C–E) that do not involve the L7 CXXC motif (Figure S9C–E), even though a CXXC motif is particularly common in TRX and PDI protein active sites. TRXs have been shown to coordinate metals,^51–53^ and genome mining for CXXC motifs reveals many associations with known redox active proteins^54^; however, CXXC motifs whose function does not appear to involve metal binding have previously been identified in multiple membrane proteins^55–57^ as now may also be the case for PfCRT.

Taken together, the results allow us to propose a model for how the L7 conformation affects the activity of PfCRT in a redox-dependent fashion (Figure 9).

**Figure 9:**
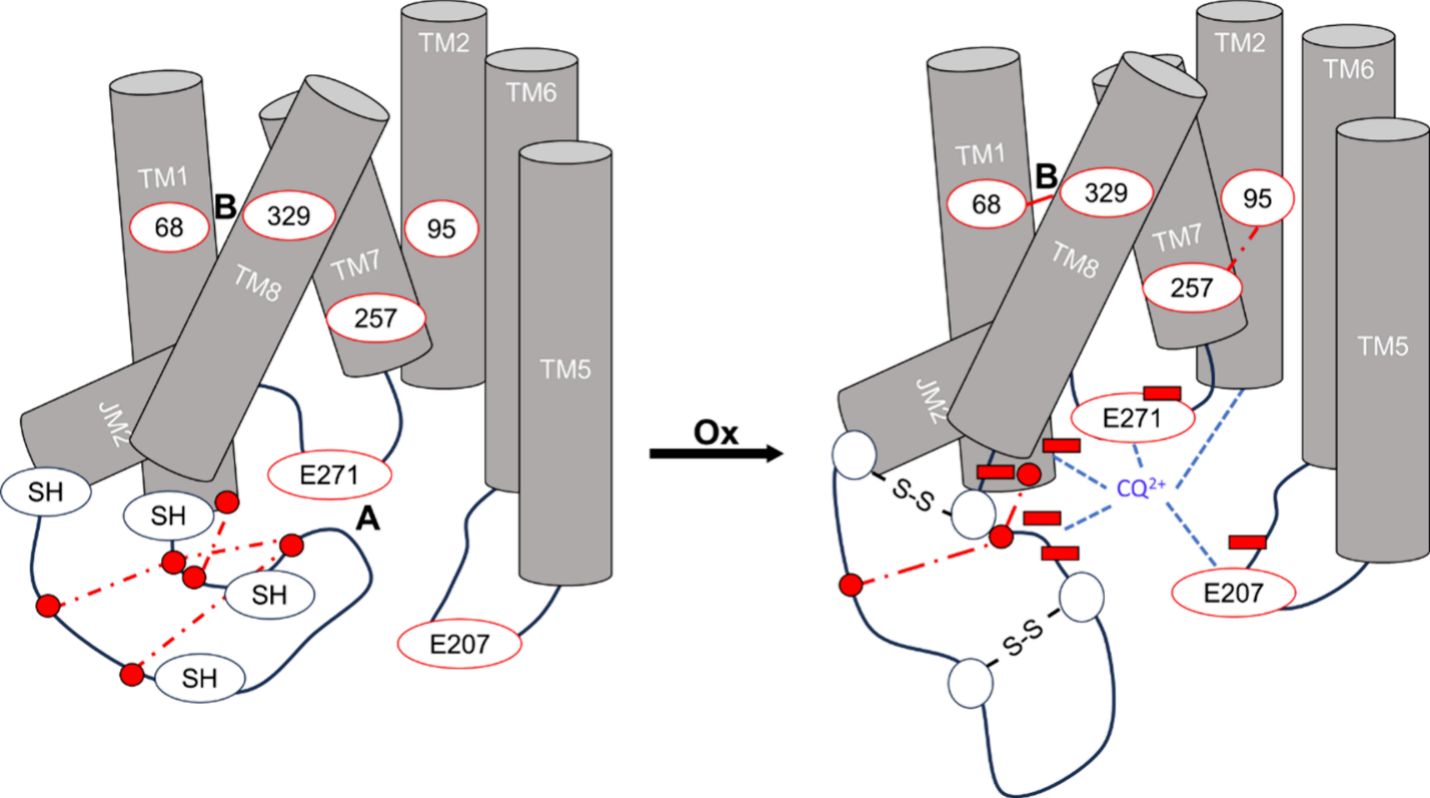
Model of L7 C redox-mediated changes. In the reduced form (left), the ‘lariat’ of L7 partially blocks access to drug binding site A. We hypothesize that this “closed” conformation is stabilized by multiple intraloop and intradrug binding site A side chain-side chain interactions, namely, K85–D311, T296–D310, E299–K307, and D310–K307 (closed red circles, dashed lines). This predicted conformation of the ‘lariat’ inhibits attraction of positively charged CQ^2+^ to site A near the DV disposed side of Dd2 PfCRT TMs. Upon oxidation, disulfide formation in L7 (open circles, S–S, right) predicts breaking of the ″closed″ conformation intraloop HBs and SBs, permitting greater access to site A. CQ^2+^ binding is further stabilized by new HBs (blue dashed lines). This change is likely transmitted to other regions of the protein, leading to other HBs being rearranged, and the resulting “open” orientation electrostatically guides CQ^2+^ to site A where it is stabilized by interactions with N84, F145, L148, E207, and E271 as previously hypothesized.^28^

Lastly, the geometric dihedral strain energy (an empirical calculation to quantify strain based upon bond dihedral angles) of the 2 L7 disulfide bonds is likely high (e.g., significantly strained; 24.6 and 44.5 kJ/mol for C289/C312 and C301/C309, respectively) relative to the average across all identified S–S within the PDB (∼10 kJ/mol) (Figure S10).^83,84^ These dihedral angles classify the disulfide bonds as ± RHHook and ± LHHook, which are associated with reversible, redox-controlled disulfides.^83,85–87^ Indeed, across our MD simulations for both the Dd2 EMMD and AFMD PfCRT structures with both L7 disulfides intact, as well as for Dd2 EMMD with a singly broken disulfide, we find that both C289/C312 and C301/C309 pairs are in these strained conformations and that their average DSE over the course of the MD calculations is ∼40 kJ/mol (Figure S10). This further supports the notion that L7 disulfide formation is reversible.

## Discussion

We find that substitution of any C (by S or A) within Dd2 PfCRT L7 fully abrogates CQ transport by PfCRT within our limits of detection, whereas mutation (to S or A) of any other of the 10 C found throughout the rest of the protein does not. Using well understood redox buffering,^58–66^ we find that PfCRT CQ transport activity is highly regulated by redox potential, being negligible at potentials < −220 mV, but ∼75% functional at potentials > −120 mV. Finally, we note that either of the 2 pairs of the 4 L7 C that form S–S can only be completely labeled with maleimide–biotin when PfCRT is fully reduced. Taken together along with MD data, these results suggest that proximal C289/C309 and C301/C312 L7 pairs of C, as suggested by the experimental cryo-EM structure of PfCRT,^1^ are indeed redox active. We hypothesize that their interconversion from free thiol to disulfide form regulates PfCRT CQ transport. This is the first example to our knowledge in which oxidation to 2 disulfides within the same loop of a transporter is required for function.

It might initially appear counterintuitive that the oxidized form of the drug resistance protein is the active drug transporting form, given the highly reducing nature of the DV. However, the redox potential of the DV as controlled by GSH is intimately tied to pH,^67–69^ and significant, large oxidizing changes in DV redox potential occur upon diffusion of CQ into the DV.^10^ The necessity of the thiolate anion as an intermediate to disulfide formation and the high p*K*_a_ of thiol reducing agents leads to increasingly lower reducing power as pH decreases. In the case of GSH, whose thiol p*K*_a_ is ∼8.7, the pseudo-standard potential shifts from −240 to −154 and −130 mV at pHs of 5.6 and 5.2, respectively.^67^ Assuming [GSSG] is near that of the cytosol, this would result in a range of redox potentials between −133 and −163 mV at pH 5.2 and −157 and −188 mV for pH 5.6 as calculated from the pH 7 midpoint potential.^67^ This would promote more PfCRT to be oxidized at any given time in CQR parasites simply due to DV pH. The p*K*_a_ of the L7 C and hence disulfide status may be further affected by positively charged lysines (K307 and K85) that are near redox active C.^70,71^

Also, the recently quantified oxidative burst promoted by drug diffusion into the DV, induces an ∼3.8 mM drop in DV GSH during 3 min exposure to plasma concentrations of drug.^10^ We calculate that at pHs 5.2 and 5.6, the redox potential then becomes ∼85–100% more oxidizing, increasing from a range of −133 to −188 mV to a range of 0 to −25 mV. This would lead to the majority of PfCRT being in its oxidized form in the presence of CQ^2+^ trapped within the more acidic DV for CQR parasites, leading to enhanced observed efflux of CQ from the DV by the redox sensitive drug transporter.^9^

Several other examples of disulfides formed in reducing cellular compartments have been identified.^72,73^ Perhaps most relevant to the malarial parasite, ROS including hydrogen peroxide, superoxide, hydroxyl, and hydroperoxyl radicals are generated during Hb catabolism within the DV^10^ and all can react with thiol to form unstable sulfenic acids, which can then rapidly react with nearby thiol to form a disulfide.^74,75^ Given the proximity of the L7 C to such ROS,^10^ it is possible that sulfenic acid modified C could readily react to form L7 disulfides *in vivo.*

Multiple redox controlled membrane proteins and transporters have been previously identified, but to our knowledge, PfCRT is the first redox regulated transporter where relevant C residue thiols are disposed to the interior of a lysosome (e.g., the specialized malarial parasite DV), and where 2 disulfides in the same loop regulate activity. For example, amino acid transport by human proton coupled amino acid transporter 1 (hPAT1)^20^ or by SNAT4 ^27^ are each gated by one extracellularly disposed disulfide bond and in both cases, reduction of the one disulfide leads to ablated transport.

Functionally important disulfide bonds have also been identified in calcium channels,^23,76^ mitochondrial TIM17,^77^ and ABCG2.^21^ ABCA1 appears to require the presence of 2 disulfides, one each in loop1 and 2,^78^ and transient receptor potential channels 1, 4, and 5 contain an extracellular disulfide necessary for dimerization and function.^79–81^ Nontransporter membrane proteins also contain thiol redox ‘switches’, including tissue factor, ADAM17, certain integrins, and TNFRSF8.^81^ The human BK channels’ affinity for FPIX heme is controlled by a SH HS/S–S interconversion found within a CXXCH motif that coordinates to heme iron^22^ and DsbD utilizes two disulfides to catalyze electron transfer.^82^

We note that redox buffering systems have been previously utilized to titrate the midpoint potential of disulfides within such proteins.^58–66^ In the case of PfCRT, our data suggest significant structural changes upon reduction of two L7 disulfide bonds at a physiologically meaningful redox potential. In the reduced form of the protein, the L7 ‘lariat’ likely occludes the opening of the CQ^2+^ binding site A, as demonstrated by a decrease in the distance between G304 (at the tip of the ‘lariat’) and residues L148, I212, and Y264 whereas when oxidized the L7 lariat allows drug access to a more ″open″ site A. Taken together, we hypothesize that increased oxidation of the L7 C leading to increased L7 S–S is a key step in increased CQ translocation out of the DV that is triggered by the oxidative burst we have measured upon drug diffusion into the DV.^10^

With this newly elucidated control mechanism, experiments aimed at identifying the natural substrate(s) of PfCRT can be reinterpreted. For example, oocyte cytosol, like all eukaryotes, ranges from −200 to 300 mV^88,89^ where S–S are less likely, and the major buffer used in oocyte-based PfCRT functional studies, ND96, contains mM amounts of divalent metal ions, which may interfere with disulfide formation. These observations may explain why PfCRT activity measured using oocyte expression systems^90^ is lower than that measured for purified PfCRT.^7^ Further studies, including live parasite transfectants, are needed to further test these and related questions.

## Conclusions

We have shown that 2 proximal C pairs (residues C289/C312 and C310/C309) in loop 7 of the PfCRT protein interconvert between reduced free thiol (SH) to oxidized disulfide (S–S) form and that a redox “switch” involving both pairs may regulate CQ^2+^ transport by PfCRT. Titrating PfCRT drug transport activity in the presence of redox buffering shows a midpoint near −140 mV, which is also near the midpoint of maleimide labeling of the four L7 C residues’ free thiol groups.

## Supplementary Material



## Notes

Figure S1: Growth of CH1305 yeast in oxidized and reduced redox buffer components; Figure S2: Ellman’s assay borohydride reduction detection of redox buffer components; Figure S3: Validation of redox potential in redox buffered growth assay; Figure S4: Experimental design for maleimide and maleimide biotin labeling; Figure S5: MB labeling using expt A and expt B conditions; Figure S6: PfCRT conformational changes in response to oxidation/reduction of L7 disulfides; Figure S7: E207–K80 distance for oxidized vs reduced Dd2 PfCRT; Figure S8: homology modeled Occ conformation of Dd2 PfCRT vs Dd2 OtV PfCRT; Figure S9: Alphafold3 and Metal3D divalent metal ion binding predictions; Figure S10: Disulfide dihedral angle and empirical dihedral strain energy of L7 C; Figure S11: C_α_ average distances; Table S1: Forward and reverse oligonucleotide primers used in this study; Table S2: concentrations of oxidized and reduced BME used in redox buffering; Table S3: Concentrations of oxidized and reduced DTT used in redox buffering; Table S4: Concentrations of oxidized and reduced TCEP used in redox buffering; Table S5: Composition of M/MB experiment A and experiment B reactions; Table S6: Redox buffered maleimide and maleimide biotin labeling experiments; Table S7: CQ transport function of C to S/A mutants relative to Dd2 PfCRT; Table S8: Quantitative densitometry and calculated # of C labeled in M/MB experiments; Table S9: Quantitative densitometry and calculated # of C labeled in M/MB experiments during redox dependent labeling calculation of redox buffer conditions (PDF)

## Notes

THE PDB ID for the CryO-EM 7G8 PfCRT structure is 6UKJ. PfCRT 7G8:W7FI62 (https://www.uniprot.org/uniprotkb/W7FI62/entry). PfCRT Dd2: F5CEB4 (https://www.uniprot.org/uniprotkb/F5CEB4/entry). TPT homodimer: B5AJT1 (https://www.uniprot.org/uniprotkb/B5AJT1/entry).

## LIST OF ABBREVIATIONS

ACTs: Artemisinin combination therapies
AMP: Ampicillin
CM: Crude membrane
CQ: Chloroquine
CQR: Chloroquine resistant
CQS: Chloroquine sensitive
CSM – URA: Complete Synthetic Media minus Uracil
Ca: α Carbon
DSE: Dihedral strain energy
DV: Digestive vacuole
EMMD: Energy minimized using the cryo-EM structure template
EV: Empty vector
FPIX: Ferriprotoporphyrin IX
GSH: Glutathione
Hb: Hemoglobin
HB: Amino acid side chain-side chain hydrogen bond
Hz: Hemozoin
iRBC: Infected red blood cell
LB: Luria broth
L7: Loop 7 of PfCRT
M: Maleimide
MB: Maleimide biotin
MD: Molecular dynamics
PfCRT: *Plasmodium falciparum* chloroquine resistance transporter
RBC: Red blood cell
Redox: Reductive/oxidative environment
RMSD: Root mean squared deviation
ROS: Reactive oxygen species
SD-URA: Synthetic dextrose – Uracil
SGR-URA: Synthetic galactose raffinose – Uracil
SHE: Standard hydrogen eectrode
YNB-AA: Yeast nitrogen base minus Amino Acids
