# Supplementary material for: Plasmodium falciparum Chloroquine Resistance Transporter (PfCRT) Is a Redox-Dependent Drug Transporter
Source: Biochemistry. 2026 Mar 11;65(7):1033–46. doi: 10.1021/acs.biochem.5c00802 (PMC13063427; doi:10.1021/acs.biochem.5c00802)
Supplement: Supplementary file 1 [file bi5c00802_si_001.pdf]

"Supporting Information"

Plasmodium falciparum Chloroquine Resistance Transporter (PfCRT) is a Redox Dependent Drug Transporter

Darius Chernitsky - Hamd[#], Rajat Roy[#], Jonas Schemm, Aubrey Schall, Mera Petros, Andreas Willems & Paul D. Roepe[*]
Dept. of Chemistry and Dept. of Biochemistry and Cellular and Molecular Biology
Georgetown University
37th and O Streets NW
Washington, DC 20057, U.S.A.

1) Figure S1 Growth of CH1305 Yeast in Oxidized and Reduced RedOx Buffer Components
2) Figure S2 Ellman's Assay Borohydride Reduction Detection of RedOx Buffer Components
3) Figure S3: Validation of RedOx Potential in RedOx Buffered Growth Assay
4) Figure S4: Experimental Design for Maleimide and Maleimide Biotin Labelling
5) Figure S5: MB labeling using expt.A and expt.B conditions
6) Figure S6: PfCRT conformational changes in response to oxidation/reduction of L7 disulfides
7) Figure S7: E207 - K80 distance for Oxidized vs Reduced Dd2 PfCRT
8) Figure S8: Homology Modelled Occ Conformation of Dd2 PfCRT vs Dd2 OtV PfCRT
9) Figure S9: Alphafold3 and Metal3D Divalent Metal Ion Binding Predictions
10) Figure S10: Disulfide Dihedral Angle and Empirical Dihedral Strain Energy of L7 C
11) Figure S11: C_α Average distances
12) Table S1 Forward and reverse oligo nucleotide primers used in this study
13) Table S2: Concentrations of Oxidized and Reduced BME used in RedOx Buffering.
14) Table S3: Concentrations of Oxidized and Reduced DTT used in RedOx Buffering.
15) Table S4: Concentrations of Oxidized and Reduced TCEP used in RedOx Buffering.
16) Table S5: Composition of M/MB Experiment A and Experiment B Reactions
17) Table S6: RedOx Buffered Maleimide and Maleimide Biotin Labelling Experiments
18) Table S7: CQ Transport Function of C to S/A Mutants Relative to Dd2 PfCRT
19) Table S8: Quantitative Densitometry and Calculated # of C Labelled in M/MB Experiments
20) Table S9: Quantitative Densitometry and Calculated # of C Labelled in M/MB Experiments During Redox Dependent Labeling
21) Calculation of Redox Buffer Conditions

[#] These authors contributed equally to this study
[*] Address correspondence to this author at roepep@georgetown.edu

Key words: drug resistance, redox, disulfide, thiol, molecular dynamic

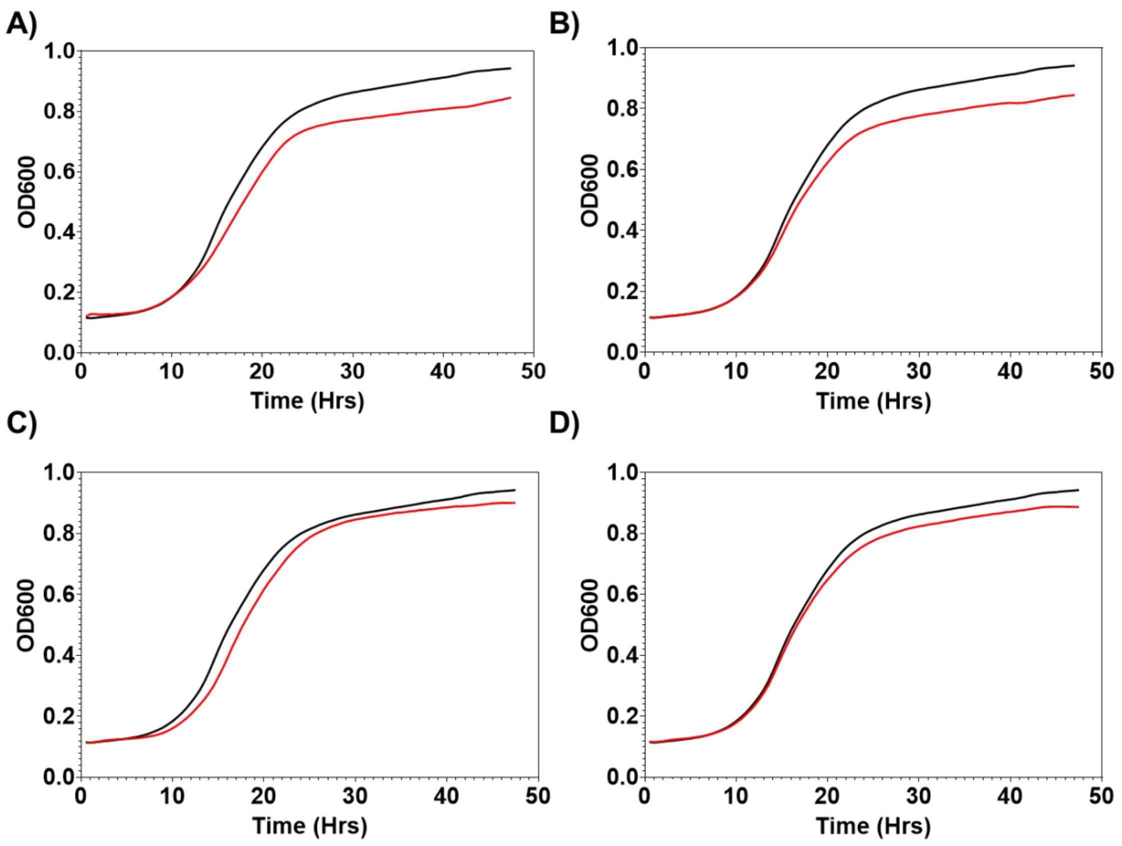

Figure S1: Concentrations of the oxidized and reduced forms of BME and DTT used here are minimally toxic to yeast during growth of CH1305 yeast. Growth curves for CH1305 yeast seeded in mid-exponential phase harboring the empty pYES2 plasmid growing in pH 7 buffered SGR-URA in the presence of 0 mM (black) and 1.5 mM thiol (red) from ß-ME (A), ßME Dimer (B), DTT (C, red indicates growth in 0.725 mM DTT, equivalent to 1.5 mM thiol), and cyclo-DTT (D).

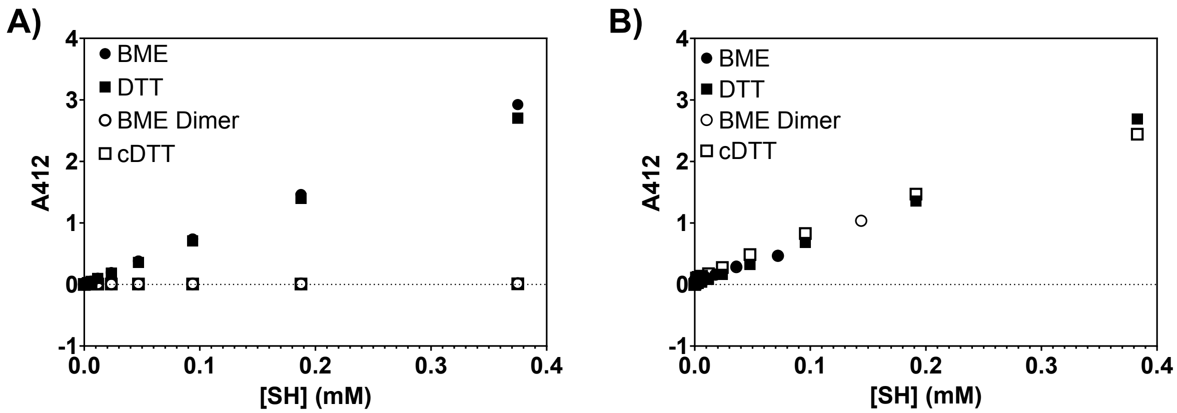

Figure S2: Validation of Modified Ellman's Assay. Ellman's assay response of ßME monomer (closed circles), DTT (closed squares), ßME dimer (open circles) and cyclo-DTT (open squares) before (A) and after (B) sodium borohydride reduction for thiol dissolved in inducing yeast media (SGR-URA). X axis corresponds to the total thiol concentration. For chemical reduction, 10 µL of redox buffered growth media supernatant was diluted to 80 uL in DI water in a 12 well basin. 20 µL of 8M Sodium Borohydride dissolved in 1.5 M NaOH and 50% MeOH:H_2O was added to each sample and incubated with light shaking at RT for 30 min. After reduction sodium borohydride was removed via spontaneous decomposition under acidic conditions. 17 µL of concentrated HCl was aliquoted into a new 12 well basin and placed on ice for 5 minutes to prevent heating and evaporation during quenching of the reaction. Samples were added slowly to the HCl, and the mixture was vortexed in an Eppendorf MixMate for ~30s to ensure the reaction was complete. 30 µL of the diluted product was added to a flat bottom 96-well plate supplemented with 1M Tris-Cl pH 8.2 and 10 µL of 10 mM DTNB for a final volume of 200 µL. The plate was allowed to incubate in the dark for 15 min and absorbance at 412 nm was then measured. Calculation of the concentrations of the oxidized forms in a given sample was done by subtracting the measured free reduced form concentration from the total (and dividing by 2). The relative stability of the thiol redox buffers in the presence of growing yeast is detailed in Figures S2 and S3.

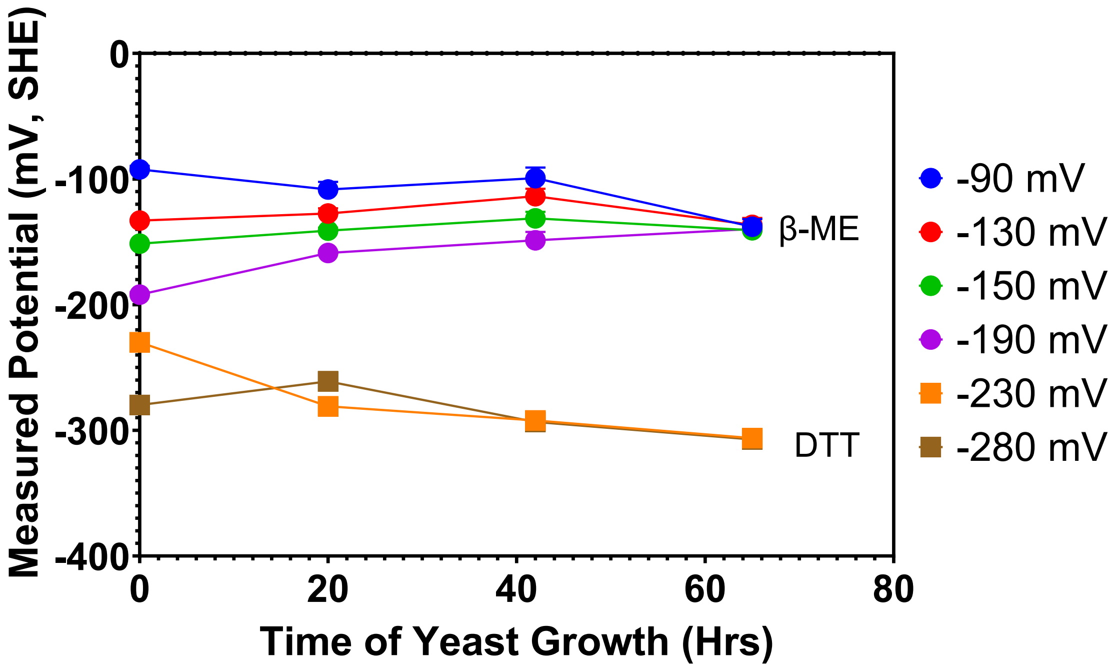

Figure S3: RedOx Buffer Stability in Redox Buffered TeCan Assay. Calculated initial pH 7 (key) vs measured RedOx potentials for growing CH1305 yeast over the course of 3 days using the modified Ellman's assay (see methods). Plotted points correspond to 2 independent experiments with each experiment consisting of 2 independent single yeast colonies and 4 determinations for each potential and time point. Error bars correspond to SEM.

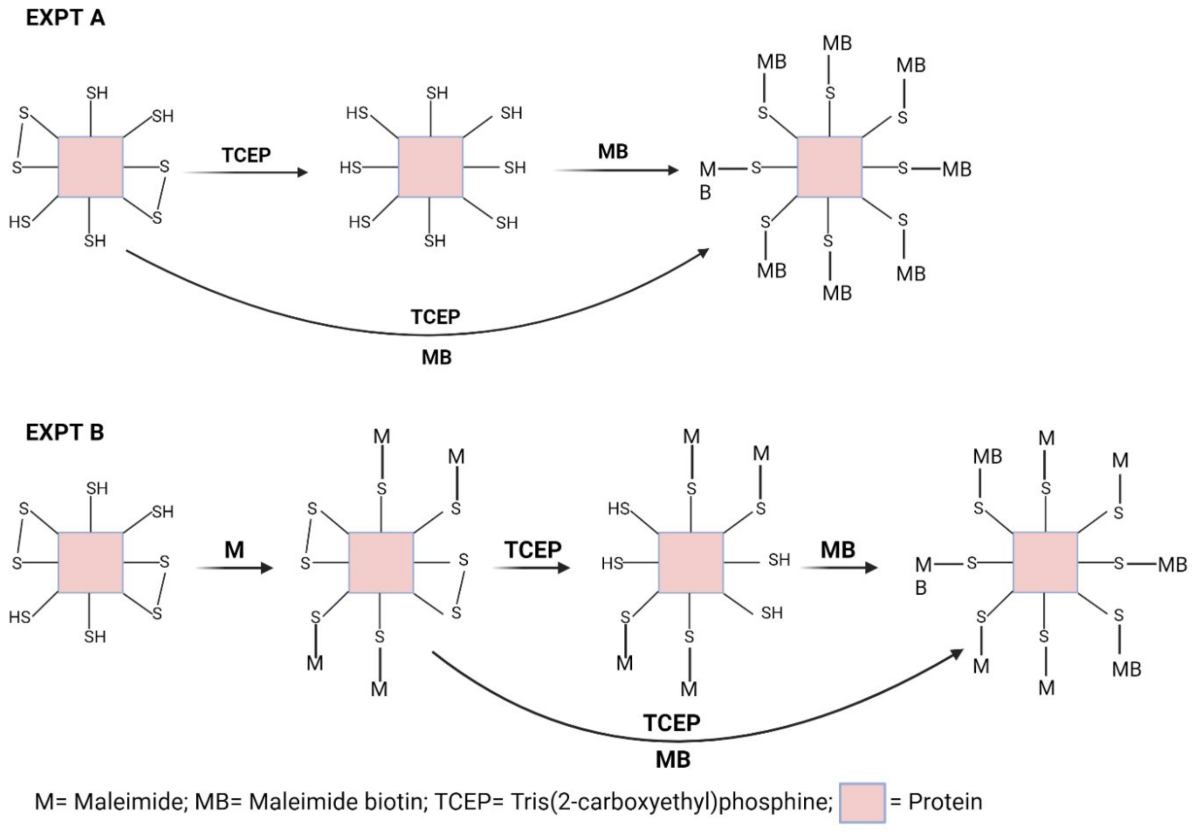

Figure S4: Cartoon of Experiment A vs Experiment B formats for MB Labeling of PfCRT protein. In “expt.A” (top) the protein was studied under fully reduced conditions, so MB was added in the presence of reducing agent TCEP, to fully expose all PfCRT SH, and the reaction continued for 4 hr. Under “expt.B” conditions (bottom), PfCRT was treated with M under ambient conditions for 4 hr. to react with (block) all free thiol available under ambient conditions with M. The protein was then reduced and labeled with MB for 4 hr. more, allowing only previously sequestered C to react with MB.

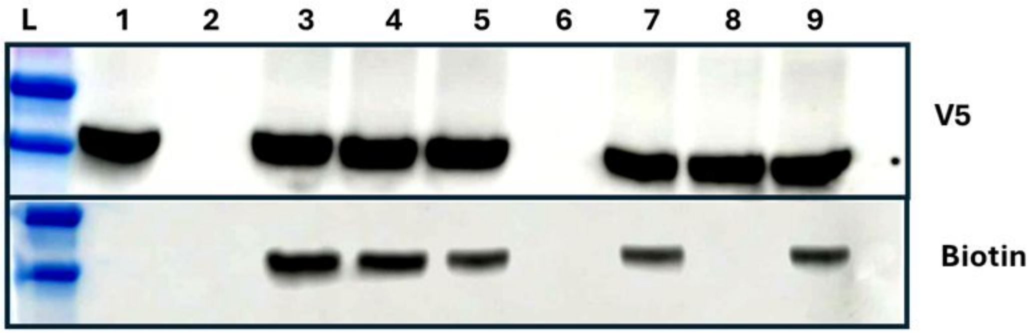

Figure S5: MB labeling for C10XS,A vs Dd2 PfCRT. V5 tag detection (top) and Biotin detection of MB (bottom) for 12xHIS-nickel bead pulldowns of PfCRT from yeast membranes harboring Dd2 PfCRT (lanes 1,3,7), no PfCRT (lanes 2 & 6) Dd2 L7S4 (lanes 4,8), or Dd2 10xC,S (lanes 5,9). Maleimide biotin (MB) and maleimide blocking (M) reaction conditions were as described for exp't. A (left, lanes 1-5) or B (right lanes 6 - 9) [Fig. S4] and consisted of a no MB negative labelling control (left, lane 1), complete reduction with TCEP followed by MB labelling ("expt. A", middle, lanes 2 - 5), or blocking with underivatized maleimide under ambient conditions followed by complete reduction with TCEP and MB labelling ("expt. B", right, lanes 6-9). Labelling ratios (shown in Table S7) were calculated from the ratio of the V5-normalized biotin band intensity to that of the band for Dd2 (e.g. 14 labelled C, lane 3, see caption to Table S7).

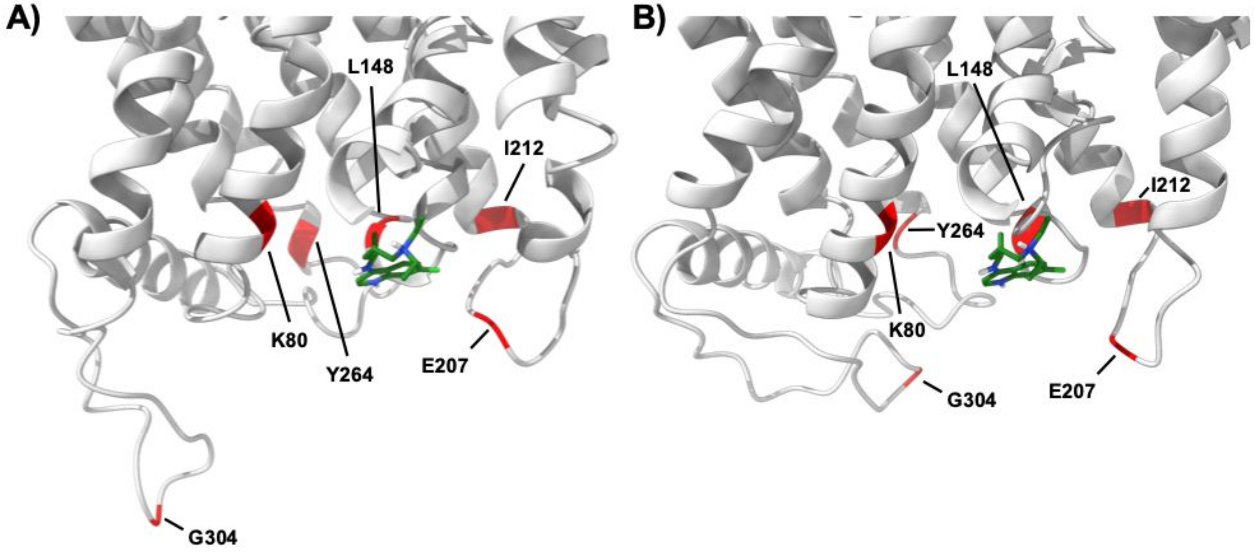

Figure S6: Visualization of regions involved in conformational changes in response to oxidation/reduction of L7 disulfides. MD minimized oxidized (left) vs reduced (right) Dd2 structures, with CQ (green) docked in drug binding site A showing the dramatic change in L7 proximity to site A upon reduction vs oxidation. Residues used for quantification of movement of the G304 containing 'lariat' of L7 (Fig. 7C; E271, L148, and Y264) as well as K80 and E207 discussed in Fig. S7, S8 are annotated and shown in red.

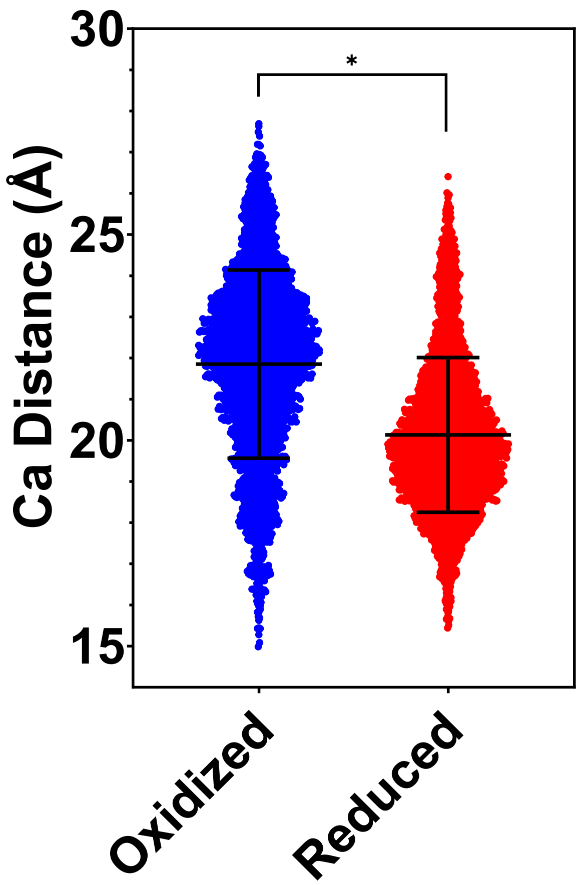

Figure S7: Change in C_α distance between E207 and K80 over the course of 3x10 ns MD calculations for reduced vs oxidized PfCRT. Scatterplot of the C_α distance between residues E207 and K80 for each MC/MD frame for oxidized (left, blue) and reduced (red, right) Dd2 EMMD structures. Error bars represent the average and SD across all frames. "*" indicates a p-value < 0.0001 by Welch's unpaired t-test. However, based on the intrinsic variability of MD atomic distance results[28], even if reproducibility satisfies the T test, we are skeptical of changes in distance < 2 Å shown by MD as documented here.

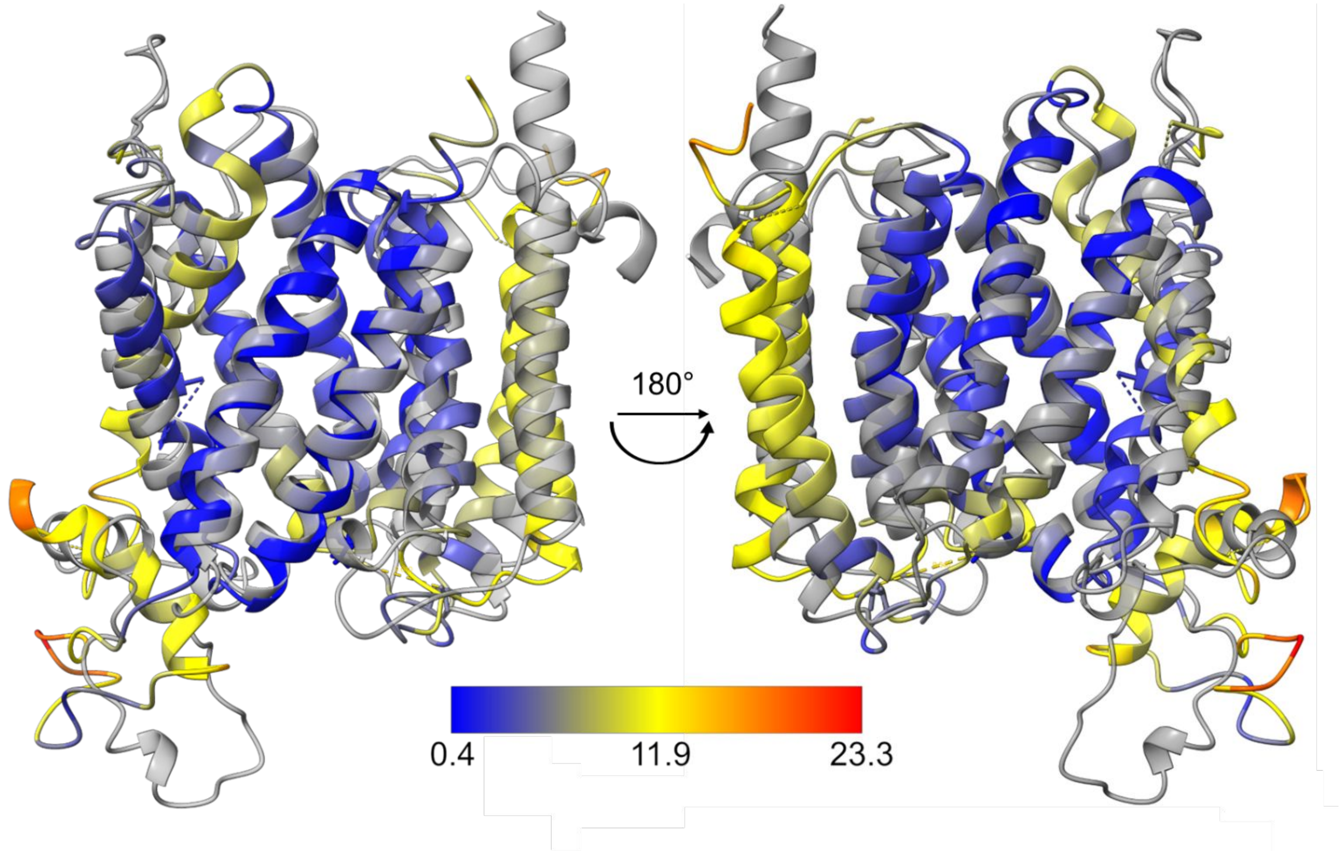

Figure S8. Overlay of homology modelled Occ Dd2 PfCRT [Occ][48] vs Dd2 EMMD (OtV) PfCRT structure derived from the 7G8 experimental cryo - EM structure described previously[28]. The minimized Occ model pdb file[48] (colored, opaque) was downloaded from Zenodo[48] and then aligned vs minimized Dd2 EMMD PfCRT[28] (grey, transparent) by ChimeraX's best chain alignment algorithm. The included color key bar denotes Cα RMSD for similarly colored residues. The average all atom RMSD as calculated using PyMol's ColorByRMSD function was 5.9 Å. Although the DV disposed end of helix 1, harboring K80, has a low Cα RMSD between 0.4 and 1 Å (blue color) revealing excellent structural similarity

between OCC and OtV in this small region, the DV disposed ends of helices 3,5,7 and 10 as well as loop 5 harboring E207 have high Cα RMSD between 5 and 10 Å (yellow). It is this movement found in the Occ homology model that forces K80 and E207 to be within permissive SB distance during MD simulation of Occ, even if they are at far greater (non SB - permissive) distances in the OtV or OtC conformations (see text). Overall, as shown (Fig. S10) nearly as many helices do not align well in the two structures as do align suggesting structural similarity between PfCRT Occ built by homology modeling to TPT vs. the Dd2 EMMD (OtV) PfCRT[1,28] structure is weak. More specifically, the original cryo EM structure of isoform 7G8 PfCRT[1] and previous work[28] to generate the Dd2 EMMD structure places E207 and K80 C_{α} >14 Å or >18 Å apart in either CQ undocked or docked Dd2 EMMD or AFMD structures, respectively, and >22 Å apart for the earlier modeled "open to cytosol" conformation for 7G8 PfCRT[1]. Hetero atom distances are ~10, >11, and >25 Å apart, respectively, all of which are much farther than the ≤ 4.0 Å hetero atom distance "cutoff" typically used to identify the proximity for forming a productive salt bridge (SB). In contrast, earlier models[48] using comparison between "open to the DV" (OtV) vs "open to the cytosol" (OtC) vs a proposed "occluded" (Occ) conformations of PfCRT place E207 and K80 as potentially involved in a dynamic SB that is visible in Occ but not in OtV or OtC. This model[48] for the Occ was built as an homology model using partial PfCRT sequence (residues 47 - 405) as input and the structure of a substrate - bound triose phosphate translocator (TPT) homodimer (PDB 5Y79) as template. In our hands, a homology model built using the same truncated PfCRT sequence (residues 47-405 shown in the 7G8 PfCRT cryo-EM structure[1]), the same TPT 5Y79 pdb file as template, and "SwissModel" homology modelling software (https://swissmodel.expasy.org/) indeed results in E207 - K80 hetero atoms ~ 2.7 Å apart after energy minimization and thus close enough to form a productive SB, but we also observe very large scale global structural changes throughout the entire remainder of the Occ protein relative to Dd2 EMMD[28] (Fig. S10, shown above) or to the 7G8 PfCRT experimental cryo - EM structure (not shown). This suggests to us that a PfCRT homology model built using a truncated PfCRT sequence vs TPT is likely unreliable, indeed the mean RMSD for PfCRT OtV [the energy minimized Dd2 PfCRT structure derived from the 7G8 PfCRT cryo-EM structure[28]] vs Dd2 PfCRT Occ, either the Occ previously constructed[48] or newly modelled here by SwissModel, is 5.9 or 5.7 Å, respectively. When the full length Dd2 PfCRT sequence is used as input vs the TPT (PDB 5Y79) structure as template, no homology model at all

can be built by us using "SwissModel" as is also mentioned[48] when those authors used different homology modelling software. Consistent with these conclusions, the TM score value when comparing the earlier Occ[48] to Dd2 EMMD[28] is .63, suggesting at least one similar fold, but also meaningful structural divergence. In contrast, neglecting N and C termini that are predicted to form a triple helical bundle named the PfCRT "zipper"[28] that is not resolved in the original 7G8 cryo-EM experimental structure, Alphafold 2 predictions for Dd2 PfCRT give a structure that is nearly identical to the Dd2 EMMD or experimental 7G8 cryo - EM structures[28] (RMSD ~ 2.9 Å) and similarly do not predict an E207 - K80 SB. Thus at this juncture, based on previously determined cryo-EM, EMMD and AFMD structures for PfCRT isoforms[28], we do not include E207/K80 as an HB or SB pair but continue to suggest that E207 is important for drug docking to site A[28] and note that both points require further study as predicted conformations of PfCRT are tested further.

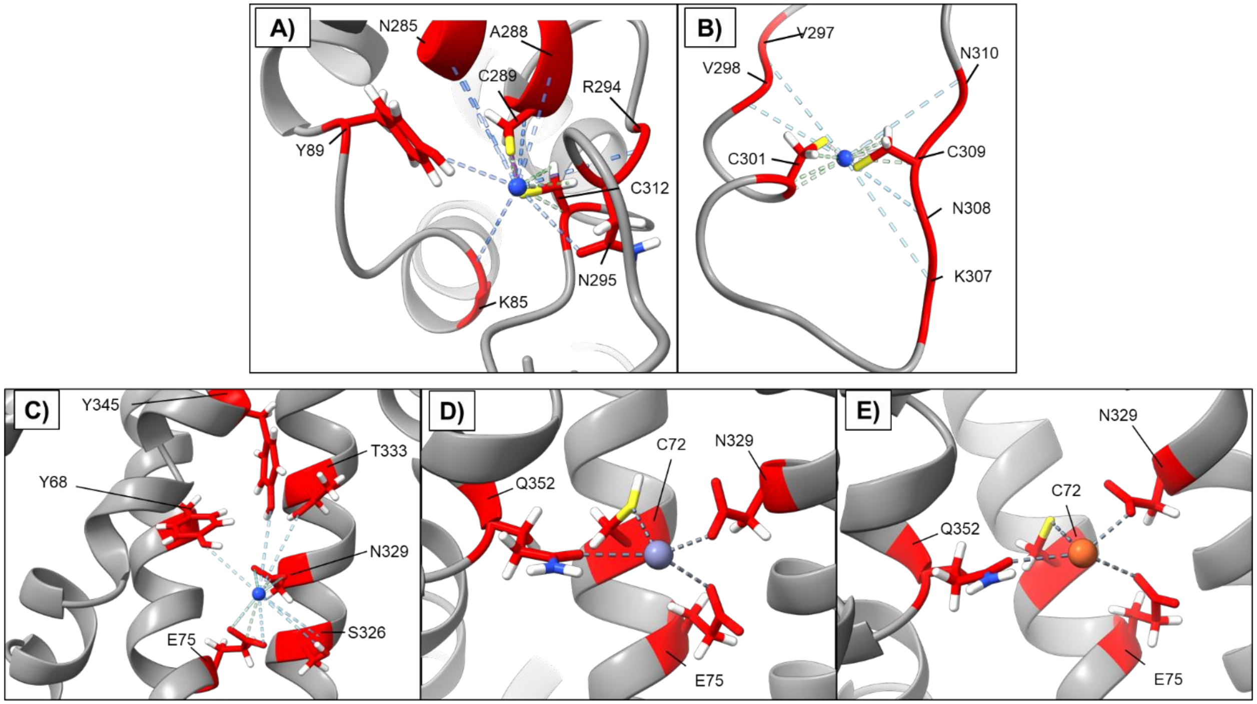

Figure S9: Putative PfCRT Zn (II) and Fe (III) metal binding sites as predicted by Metal3D and AlphaFold3 for Dd2 PfCRT. Low probability (20%) Zn (II) binding sites at L7 289-312 (A) and 301-309 (B) C as predicted by Metal3D. A higher probability (26%) Zn (II) site (C) was identified by Metal3D near putative site B. AlphaFold3 also identified Zn (II) (D) and Fe (III) sites (with pLDTT between 90 and 70) that similarly did not involve L7 C. Dashed lines indicate contacts, clashes, metal coordination, and HBs as determined by UCSF ChimeraX or Chimera.

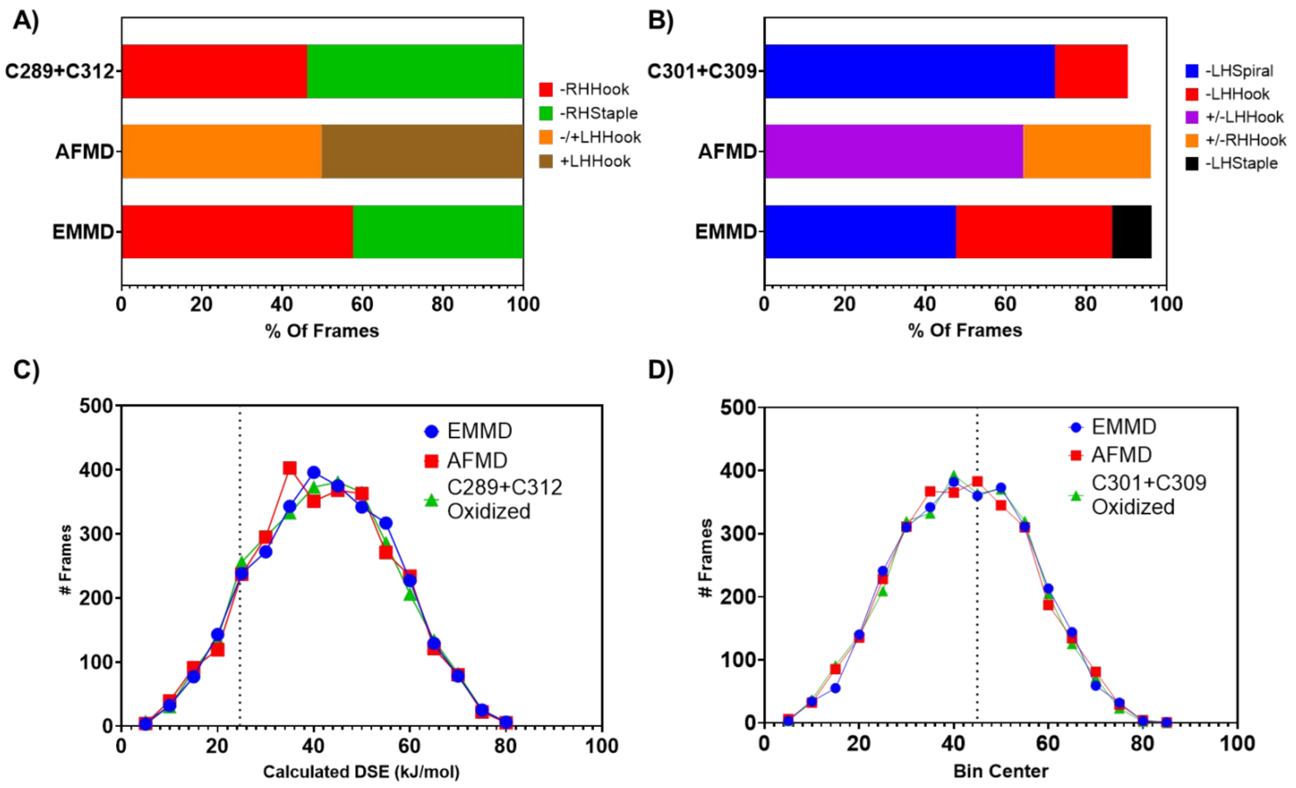

Figure S10: Geometric orientation and dihedral strain energy of L7 disulfides over the course of MD simulations. The geometric classification of L7 disulfides (A) C289-C312 and (B) C301-C309 as described by the 5 dihedral angles[83] during MD simulations for either Dd2 EMMD or AFMD[28] oxidized structures, as well as the individual C pairs as reduced SH (C289-C312 = S-S [A] or C301-C309 = S-S [B]) which appear for greater than 10% of frames. Also shown are histograms of the calculated Dihedral Strain Energy (DSE)[83,84] from the same calculations for the (C) C289-C312 disulfide and (D) C301-C309 disulfide. Dark dashed line indicates

the calculated DSE for the entire 7G8 CryoEM structure. The average DSE of all disulfides in the PDB was found to be ~10 kJ/mol[93] making both L7 PfCRT disulfides significantly more strained.

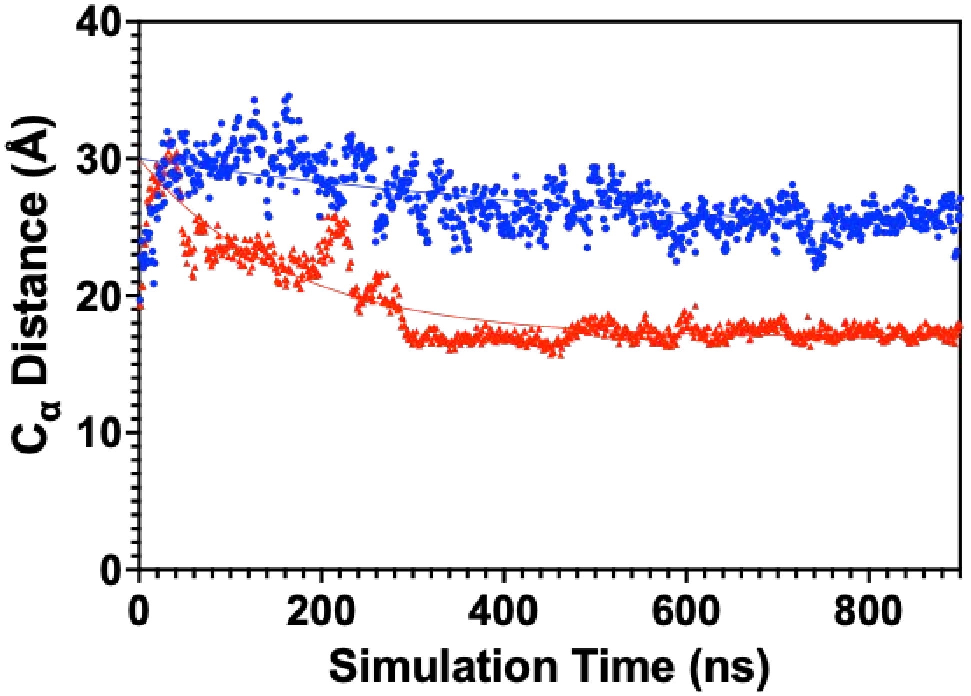

Figure S11

Caption: Average distances between G304 C_α and the C_α of 3 residues at the DV disposed ends of helices 3, 6, and 7 (L148, I212, Y264, see Fig. 7C) for 3x1 μs MD simulations of oxidized (blue) vs reduced (red) Dd2 PfCRT. Each symbol (closed blue circles vs closed red triangles) represents the average G304 C_α distance to the 3 other residue C_α computed for each frame of a VMD file that

is 900 nsec of the average trajectory of the MD simulation (see methods). Exponential curve fits to these data (superimposed thin lines) show that the MD simulated average difference in G304 C_{α} distance for oxidized vs reduced PfCRT relative to residues that line the DV drug pore opening plateaus near ~ 8 Å.

Table S1: Forward and reverse oligo nucleotide primers used in this study. Yeast optimized *pfcrt* encoding an improved C-terminal tag system that encodes a Tobacco Etch Virus (TEV) protease site between V5 tag and 12xHIS tags (top) was used as the mutagenic template. Mutagenic oligonucleotides used to create 4C L7, 2C L7, or 1C L7 to S or A, and all other possible PfCRT C to S and A substitutions are also shown. All C mutants were ultimately created on the pYES2 PfCRT Dd2-V5_TEV_12xHIS plasmid backbone, with all L7 C to S substitutions also created on a pYES PfCRT HB3-V5_TEV_12xHIS template.

Mutation(s)	Forward	Reverse	Mutation(s)	Forward	Reverse
TEV	CTTCCAGGGCGGTCACCCATTCGAAGGT	TACAGGTTCTCTTGAGTGATGATACTGTCTACG	C139S	GTTAGACGCTTCCAGCGTCATCT	ATAGAGATTGCGAAGAATTGC
6xHIS	CATCACCATCATCACCATCACCATTGACTC	GTGATGATGATGACCGGTGCCCTGGAA	C171S	CATGTTCTTCTCCTTCCTGATCTTG	TTGATTGGAATTGATAGTTGC
289S	CGGTTTCGCCTTCCTGTTCTTAG	TTCTTGATGTTGGTCCAGATTTC	C225S	GATCCCTGTCTCTTTCTCCAACATG	AGAGAGGATATCAGGACTAG
301S	TGTCGAGAACTTCGGTTTGGGCA	ACTGTGTTCCTTCCTAAGAACAG	C258S	GTTCACTTCATCCTTAATCCTGC	AGTTGGAAGAAGCTAAC
309S	GGCCAAGTTGTTCGACGACTGTG	ATGCCCAAACCGCAG	C328S	CTTCTCCATCTCTGATAACCTGATC	AATGAGAACAAGGCGAATG
312S	GTGCGACGACAGTGACGGCGCTT	AACTTGGCCATGCCCAAACC	C350S	TATTGTGAGTTCCATCCAGGGGC	GTGTAAGTCATGGTGGAG
301+309A	TGGCCAAGTTGGCTGACGACTGTGACC	TGCCCAAACCAGCGTTCTCGACAACTGTGTTC	C40+44A	GCAAGGCTGCTCACGTCTTCAAGC	CCAAAGCGCTACCTCCACCCAATC
289 + 312S	CTGCGGTTTGGGCATGGCCAAGTTGTGCGACGACAGTGACGGCGCTTGG	TTCTCGACAACTGTGTTCCTTCCTAAGAACAGGCTGGCGAAACCGTTCTTG	C40A	TGGAGGTAGCGCTTTGGGCAAGTG	CCCAATCTGGAACCG
301 + 309S	ATGGCCAAGTTGAGCGACGACTGTGAC	GCCCAAACCGCTGTTCTCGACAACTGTG	C44A	TTTGGGCAAGGCTGCTCACGTCTTC	CAGCTACCTCCACCC
289A	CGGTTTCGCCGCTCTGTTCTTAGG	TTCTTGATGTTGGTCCAG	C72A	CCTGTCAGTTGCCGTGATCGAAAC	TAGATGATGGACAGGATGTAG
301A	TGTCGAGAACGCTGGTTTGGGCATG	ACTGTGTTCCTTCCTAAG	C101A	CAACTTCATCGCTATGATCATGTTCTTCATC	TGGGTTTCACTAGTCAC
309A	GGCCAAGTTGGCTGACGACTGTGAC	ATGCCCAAACCGCAG	C139A	GTTAGACGCTGCTAGCGTCATCTTG	ATAGAGATTGCGAAGAATTG
312A	GTGCGACGACGCTGACGGCGCTTG	AACTTGGCCATGCCC	C171A	CATGTTCTTCGCTTTCCTGATCTTGAGG	TTGATTGGAATTGATAGTTGC

C40S + C44S	GGCAAGAGTGCTCACGTCTTCAAG	CAAACTGCTACCTCCACCCAATC
C40S	TGGAGGTAGCAGTTTGGGCAA	CCCAATCTGGAACCGTTTC
C44S	TTTGGGCAAGAGTGCTCACGTCT	CAGCTACCTCCACCCAATC
C72S	CCTGTCAGTTTCCGTGATCGAAAC	TAGATGATGGACAGGATGTAG
C101S	CAACTTCATCTCCATGATCATGTTC	TGGGTTTCACTAGTCAC
C225A	GATCCCTGTCGCTTTCTCCAACATGAC	AGAGAGGATATCAGGAC
C258A	GTTCACTTCAGCTTTAATCCTGCCAGTTTAC	AGTTGGAAGAAGCTAAC
C328A	CTTCTCCATCGCTGATAACCTGATCACC	AATGAGAACAAGGCGAATG
C350A	TATTGTGAGTGCTATCCAGGGGCC	GTGTAAGTCATGGTGG

Table S2: Set potentials (given against the standard hydrogen electrode [SHE]), corresponding reaction quotients, and concentrations of reduced and oxidized β-ME used for RedOx buffering of PfCRT function and MB labelling, with the total concentration of BME monomer units being 1.5 mM.

Calculated pH 7 Potential (mV | SHE)	Q	[ßME] (µM)	[ßME Dimer] (µM)
-39.98	4.8E-08	6.0	747.0
-64.86	3.2E-07	15.5	742.2
-89.89	2.2E-06	40.1	729.9
-114.38	1.4E-05	100.3	699.8
-129.67	4.6E-05	175.3	662.3
-139.81	1.0E-04	250.9	624.5
-149.70	2.1E-04	351.3	574.3
-164.86	6.9E-04	566.2	466.9
-190.28	4.8E-03	1045.4	227.3
-215.05	3.2E-02	1380.9	59.6
-239.95	2.2E-01	1479.7	10.2

Table S3: Set potentials (given against the standard hydrogen electrode [SHE]), corresponding reaction quotients, and concentrations of reduced and oxidized DTT used for RedOx buffering of PfCRT function, with the total concentration of DTT units being 1.5 mM

Calculated pH 7 Potential (mV | SHE)	Q	[DTT] (μM)	[Cyclo-DTT] (μM)
-218.80	2.01E-04	0.3	1499.7
-229.87	4.68E-04	0.7	1499.3
-239.82	1.00E-03	1.5	1498.5
-249.92	2.17E-03	3.3	1496.7
-259.98	4.70E-03	7.0	1493.0
-279.85	2.15E-02	31.6	1468.4
-299.83	9.93E-02	135.5	1364.5
-320.02	4.66E-01	476.6	1023.4

Table S4: Set potentials (given against the standard hydrogen electrode [SHE]), corresponding reaction quotients, and concentrations of reduced and oxidized TCEP used for RedOx buffered MB labelling, with the total concentration of TCEP reducing units being 10 mM.

Calculated pH 7 Potential (mV | SHE)	Q	[TCEP] (μM)	[TCEPO] (μM)
-150	8.02E-06	0.08	9980
-160	1.80E-05	0.18	9980
-170	4.21E-05	0.42	9980
-180	9.82E-05	0.98	9980
-190	2.30E-04	2.3	9980
-200	5.33E-04	5.32	9980
-220	2.83E-03	28.2	9960
-240	1.52E-02	149.4	9840
-260	8.01E-02	740	9240
-300	2.30E+00	6960	3020
-350	1.52E+02	9920	65.2
-403	1.25E+04	9980	0.8

Table S5: Reagent mixtures for M and MB experiments A and B labeling of PfCRT.

Sample	CM
Avg Conc. (μg/μL)	3
Volume (uL)	200
Total Protein (mg)	0.6
Total protein (mmoles)	0.000012
Maleimide/Maleimide Biotin (mmoles)	0.000156
TCEP (mmoles)	0.00078
Total Volume (mL)	1
Total protein (mM)	0.012
Maleamide/ Maleamide Biotin (mM)	0.156
TCEP (mM)	0.78

Table S6: Reagent mixtures for maleimide labeling experiments at different redox potentials (Fig. 6) where ßME was used as the redox buffer. The amount of M/MB was increased to compensate for potential side reactions with ∫ME thiols.

ID	RedOx Potential (mV)	10x [BME Stock] mM	10x [BME Dimer] Stock mM	Vol BME (uL)	Vol Dimer (uL)	Total volume (mL)	Maleimide/Maleimide Biotin(M)
1	-40	1	100	24	28.8	0.4	0.00021
2	-65	10	100	6.2	29.6	0.4	0.00031
3	-90	10	100	16	29	0.4	0.00055
4	-115	100	100	4	27.8	0.4	0.00115
5	-130	100	100	7	26.4	0.4	0.0019
6	-140	100	100	16	24.8	0.4	0.00415

7	-150	100	100	14	22.8	0.4	0.00365
8	-166	100	100	22.6	18.6	0.4	0.0058
9	-190	100	100	41.6	9	0.4	0.01055
10	-215	100	10	45.2	23.8	0.4	0.01145
11	-240	100	1	59	40.4	0.4	0.0149

Table S7: List of Dd2 PfCRT mutants used in this study, location of mutations relative to the Dd2 PfCRT primary sequence, and percent of Dd2 PfCRT function for the mutant assayed as described. All mutants were created on the pYES2 PfCRT Dd2-V5-tev-12XHis (-Vt12H) plasmid backbone, with mutants denoted with a * indicating the substitution was also made on a pYES2 PfCRT HB3-Vt12H background. All functional data were from analysis of a minimum of 3 independent yeast colonies expressing the mutant to similar levels as observed for Dd2 PfCRT yeast. -- indicates CQ transport was < 10% that of Dd2. N/D indicates not assayed.

Name	40	44	72	101	139	171	225	258	289	301	309	312	328	350	Percent of Dd2 Function	
															S	A
C289S/A	C	C	C	C	C	C	C	C	**S/A**	C	C	C	C	C	--	--
C301S/A	C	C	C	C	C	C	C	C	C	**S/A**	C	C	C	C	--	--
C309S	C	C	C	C	C	C	C	C	C	C	**S**	C	C	C	--	N/D
C312S	C	C	C	C	C	C	C	C	C	C	C	**S**	C	C	--	N/D
C289+312S/A*	C	C	C	C	C	C	C	C	**S/A**	C	C	**S/A**	C	C	--	--
C301+309S/A*	C	C	C	C	C	C	C	C	C	**S/A**	**S/A**	C	C	C	--	--
L7S/A4*	C	C	C	C	C	C	C	C	**S/A**	**S/A**	**S/A**	**S/A**	C	C	--	--
C44S/A	C	**S/A**	C	C	C	C	C	C	C	C	C	C	C	C	75.3 ± 9.4	74 ± 10.9
C72S/A	C	C	**S/A**	C	C	C	C	C	C	C	C	C	C	C	93.5 ± 2.8	102.8 ± 3.1
C101S/A	C	C	C	**S/A**	C	C	C	C	C	C	C	C	C	C	57.7 ± 10.9	69.6 ± 4.6
C139S/A	C	C	C	C	**S/A**	C	C	C	C	C	C	C	C	C	66.5 ± 10	74.9 ± 2.2
C171S/A	C	C	C	C	C	**S/A**	C	C	C	C	C	C	C	C	44.4 ± 5.4	78.3 ± 6.1
C225S/A	C	C	C	C	C	C	**S/A**	C	C	C	C	C	C	C	91.7 ± 2.8	86.7 ± 5.8
C258S/A	C	C	C	C	C	C	C	**S/A**	C	C	C	C	C	C	90.3 ± 7.9	89.9 ± 2
C328S/A	C	C	C	C	C	C	C	C	C	C	C	C	**S/A**	C	61.3 ± 4.8	72.6 ± 1.7
C350S/A	C	C	C	C	C	C	C	C	C	C	C	C	C	**S/A**	111.3 ± 7.3	101.2 ± 4.4
1xC, S/A	**S/A**	C	C	C	C	C	C	C	C	C	C	C	C	C	91.5 ± 3.3	95.3 ± 4.6
2xC, S/A	**S/A**	**S/A**	C	C	C	C	C	C	C	C	C	C	C	C	81.4 ± 2.7	87.3 ± 4.4
3xC, S/A	**S/A**	**S/A**	**S/A**	C	C	C	C	C	C	C	C	C	C	C	85.2 ± 5.7	70.2 ± 7.9
4xC, S/A	**S/A**	**S/A**	**S/A**	**S/A**	C	C	C	C	C	C	C	C	C	C	74.4 ± 3.7	54.6 ± 3
5xC, S/A	**S/A**	**S/A**	**S/A**	**S/A**	**S/A**	C	C	C	C	C	C	C	C	C	48.5 ± 6.2	45.9 ± 5.5
6xC, S/A	**S/A**	**S/A**	**S/A**	**S/A**	**S/A**	**S/A**	C	C	C	C	C	C	C	C	30.6 ± 4.5	48 ± 3.1
7xC, S/A	**S/A**	**S/A**	**S/A**	**S/A**	**S/A**	**S/A**	**S/A**	C	C	C	C	C	C	C	29.1 ± 0.5	44.5 ± 12.8
8xC, S/A	**S/A**	**S/A**	**S/A**	**S/A**	**S/A**	**S/A**	**S/A**	**S/A**	C	C	C	C	C	C	27.7 ± 2.1	49.1 ± 3.9
9xC, S/A	**S/A**	**S/A**	**S/A**	**S/A**	**S/A**	**S/A**	**S/A**	**S/A**	C	C	C	C	**S/A**	C	35.9 ± 0.03	53.3 ± 1.6
10xC, S/A	**S/A**	**S/A**	**S/A**	**S/A**	**S/A**	**S/A**	**S/A**	**S/A**	C	C	C	C	**S/A**	**S/A**	28.2 ± 0.7	55.4 ± 2

Table S8: Quantitative densitometry of M/MB labelling (Figures 4, 6, and S5). V5 and Biotin band intensities were extracted from membrane images via ImageJ as described in methods. The V5 intensity was used to normalize MB intensities to the amount of PfCRT in the crude membrane sample. The V5 normalized biotin intensity of Dd2 was set to 1, and sample intensities were then scaled to Dd2. The # of C was calculated using all 14 C in Dd2 are labelled in Expt. A. Mean and error correspond to the average +/- S.D. for 3 independent experiments involving 3 separate yeast growths, 3 separate membrane purifications, 3 separate labelling reactions and pulldowns, and 3 separate western blots for each sample.

Lane No.	PfCRT	Biotin/V5 Intensity Normalized to Dd2	#C Labelled
Fig 4 lane1	Dd2	1	14
Fig 4 lane2	Dd2 L7S4	0.75 ± 0.06	10.5 ± 0.84
Fig 4 lane3	Dd2 C289+312S	0.87 ± 0.05	12.18 ± 0.70
Fig 4 lane4	Dd2 C301+309S	0.86 ± 0.04	12.04 ± 0.56
Fig 4 lane5	Dd2	0.28 ± 0.06	3.92 ± 0.84
Fig 4 lane6	Dd2 L7S4	0	0
Fig 4 lane7	Dd2 C289+312S	0.13 ± 0.03	1.82 ± 0.42
Fig 4 lane8	Dd2 C301+309S	0.12 ± 0.04	1.68 ± 0.56

Fig S5 lane1	Dd2 (no MB control)	0	0
Fig S5 lane2	no PfCRT control	0	0
Fig S5 lane3	Dd2	1	14
Fig S5 lane4	Dd2 L7S4	0.75 ± 0.06	10.5 ± 0.84
Fig S5 lane5	Dd2 10xC,S	0.27 ± 0.07	3.78 ± 0.98
Fig S5 lane6	EV	0	0
Fig S5 lane7	Dd2	0.28 ± 0.06	3.92 ± 0.84
Fig S5 lane8	Dd2 L7S4	0	0
Fig S5 lane9	Dd2 10xC,S	0.29 ± 0.03	4.06 ± 0.42

Table S9: Quantitative densitometry of M/MB labelling shown in Fig. 6 vs buffered redox potential. V5 and Biotin band intensities were extracted from membrane images via ImageJ as described, V5 intensity was used to normalize all MB intensities to the amount of PfCRT in the crude membrane sample, which varied only slightly (see Fig. 4, 6). The V5 normalized biotin intensity of Dd2 via Expt.B was set to 1, and sample intensities were then scaled vs Dd2. The # of C was calculated knowing all 4 C for Dd2 in Expt. B were labelled (lane 12, Fig. 6). Mean and error correspond to the average +/- S.D. for 3 independent experiments involving 3 separate yeast growths, 3 separate membrane purifications, 3 separate labelling reactions and pulldowns, and 3 separate western blots for each sample.

Lane No.	Potential (mV)	Biotin/V5 intensity normalized to Dd2	#C labelled
1	-40	0.00 ± 0.01	0.00 ± 0.04
2	-65	0.01 ± 0.01	0.04 ± 0.04
3	-90	0.03 ± 0.03	0.12 ± 0.12
4	-115	0.04 ± 0.04	0.16 ± 0.16
5	-130	0.03 ± 0.03	0.12 ± 0.12
6	-140	0.23 ± 0.05	0.92 ± 0.20
7	-150	0.36 ± 0.02	1.44 ± 0.08
8	-166	0.44 ± 0.09	1.76 ± 0.36
9	-190	0.45 ± 0.03	1.80 ± 0.12
10	-215	0.62 ± 0.03	2.48 ± 0,12
11	-240	0.78 ± 0.03	3.12 ± 0.12

Calculation of Redox Buffer Conditions: Under non-standard conditions, the redox potential for any ratio of an oxidant and reductant pair is given by the Nernst equation, where *E* is the non-standard potential, *R* is the ideal gas constant, *T* is the temperature of the system, *z* is the number of electrons transferred during the reaction, *F* is Faradays constant, *Q* is the reaction quotient of the reduction, and E_0 is the difference in potential between the standard hydrogen electrode (SHE) [1M H+ in solution, 1 atm H_2 gas equilibrated with the solution at the platinum electrode-solution interface) vs the given redox pair at 1M concentration and STP (eq. 1).

$$E = E_0 - \frac{RT}{zF} \ln (Q) \tag{1}$$

As SHE conditions are not always able to be achieved for biologically relevant redox couples, a pseudo-standard potential, E_7', is used and defined as the potential at pH 7 with *Q* equal to 0 (eq. 2)

$$E = E_7' - \frac{RT}{zF} \ln (Q) \tag{2}$$

Given a desired potential, the corresponding reaction quotient can be derived from eq. 2, yielding eq 3.

$$Q = e^{-\frac{E-E_7'}{RT/zF}} \tag{3}$$

One way to clamp the redox potential at a given value is to use the GSH/GSSG pair, but as GSH and GSSG have been suggested as putative substrates of PfCRT[35–38] different redox buffer components were chosen. BME and DTT were chosen for our titrations as they allow for a range of potentials between -40 mV and -320 mV to be achieved at reasonable concentrations of each component, and both the oxidized and reduced components are non-toxic to growing *Sc* yeast up to 1.5 mM total thiol (Figure S1). The non-thiol reducing agent TCEP was utilized as a third redox buffer system for maleimide-biotin labelling of PfCRT cysteine thiol groups vs potential. The oxidation reactions of these reductants are shown below in scheme 1.

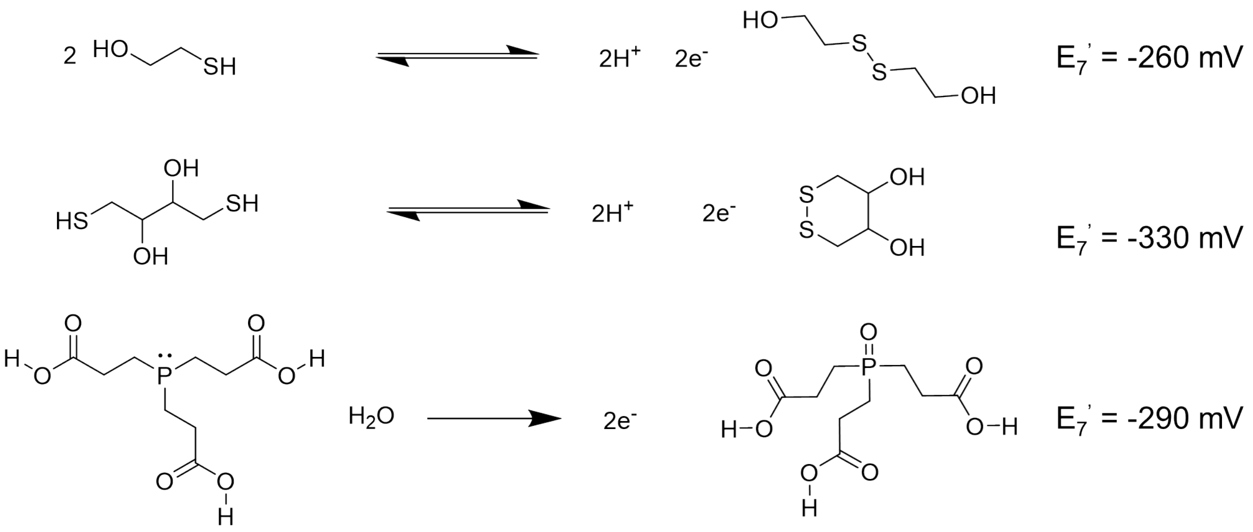

Scheme 1: Pseudo-standard potentials at pH 7 for the oxidation of β-mercaptoethanol (BME) monomer to 2-hydroxyethyl disulfide (BME-Dimer, top)[39], dithiothreitol (DTT) to trans-4,5-dihydroxy-1,2-dithiane (Cyclo-DTT, middle)[39], and Tris(2-carboxyethyl)phosphine (TCEP) to Tris(2-carboxyethyl)phosphine Oxide (TCEPO, bottom).[40]

The oxidation of ßME consists of the reaction of 2 ßME monomers to form 1 ßME dimer, with Q being given by eq 4.

$$Q_{ßME} = \frac{[ßME]^2}{[ßME\ Dimer]} \tag{4}$$

Note reduction of ßME to ßME-dimer is a net 2 electron transfer. With calculated *Q* at a desired *E*, knowing the desired total concentration of thiols, the concentration of ßME monomer can be calculated by solving the quadratic eq 4, yielding eq 5:

$$[Monomer] = \frac{1}{4}\left(\sqrt{Q} \times \sqrt{8[Total\ Monomer] + Q} - Q\right) \tag{5}$$

Given the concentration of the monomer, the concentration of the dimer (oxidized) form needed for a given potential is then derived by eq 6:

$$[Dimer] = \frac{[Total\ Monomer] - [Monomer]}{2} \tag{6}$$

The oxidation of DTT and TCEP are also net 2-electron transfers, however only 1 DTT or TCEP molecule is needed to form the oxidized form (cyclo-DTT or TCEP-O, respectively). Therefore, at a desired potential with a fixed total concentration of DTT and the calculated Q for that potential, the concentration of the reduced form is given by eq 7.

$$[Reduced] = \frac{Q[Total]}{Q+1} \tag{7}$$

The concentration of the oxidized form is then given by eq 8.

$$[Oxidized] = [Total] - [Reduced] \tag{8}$$

All concentrations of ßME monomer and ßME dimer, DTT and Cyclo-DTT, as well as TCEP and TCEP-O used for assessment of function and biotin-maleimide labelling at different redox potentials are given in Tables S1, S2, and S3, respectively.
